# Supplementary material for: ATP6V0A1-dependent cholesterol absorption in colorectal cancer cells triggers immunosuppressive signaling to inactivate memory CD8+ T cells
Source: Nat Commun. 2024 Jul 6;15:5680. doi: 10.1038/s41467-024-50077-7 (PMC11227557; doi:10.1038/s41467-024-50077-7)
Supplement: Supplementary file 1 — Supplementary Information [file 41467_2024_50077_MOESM1_ESM.docx]

**SUPPLEMENTARY FIGURES and FIGURE LEGENDS**


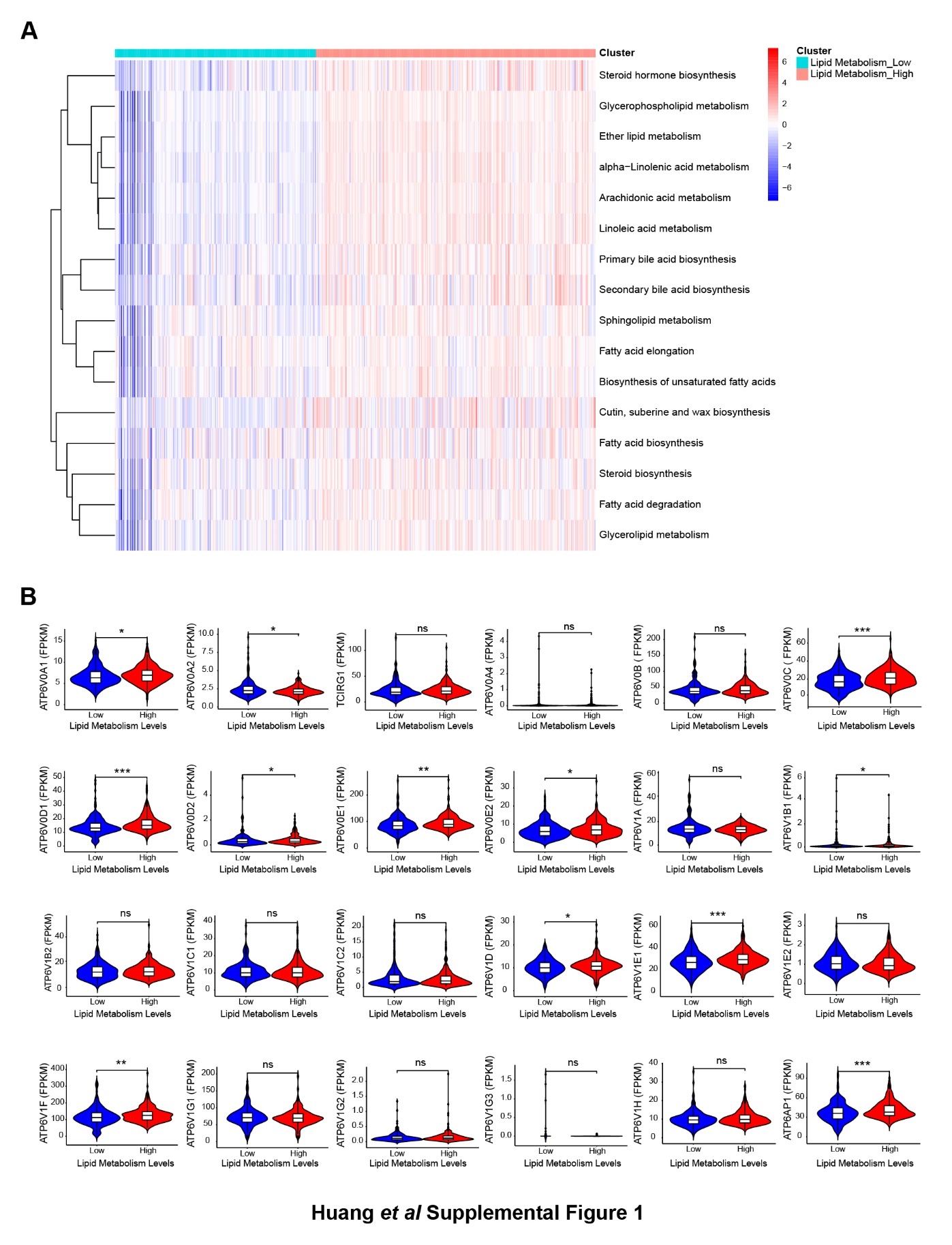


**Supplementary Fig. 1 ATP6V0A1 is positively correlated with enhanced lipid metabolism in CRC.** **A**, The 471 TCGA-COAD samples were divided into two groups based on low lipid metabolism (n=192) and high lipid metabolism (n=279), respectively, as described in the methods, and their transcriptomics-based scores of individual lipid metabolism pathways were shown in a heat map. **B**, The expression levels of different V-ATPase subunits were compared between CRC samples with high or low lipid metabolism, respectively. Statistical significance was determined using the Wilcoxon test.


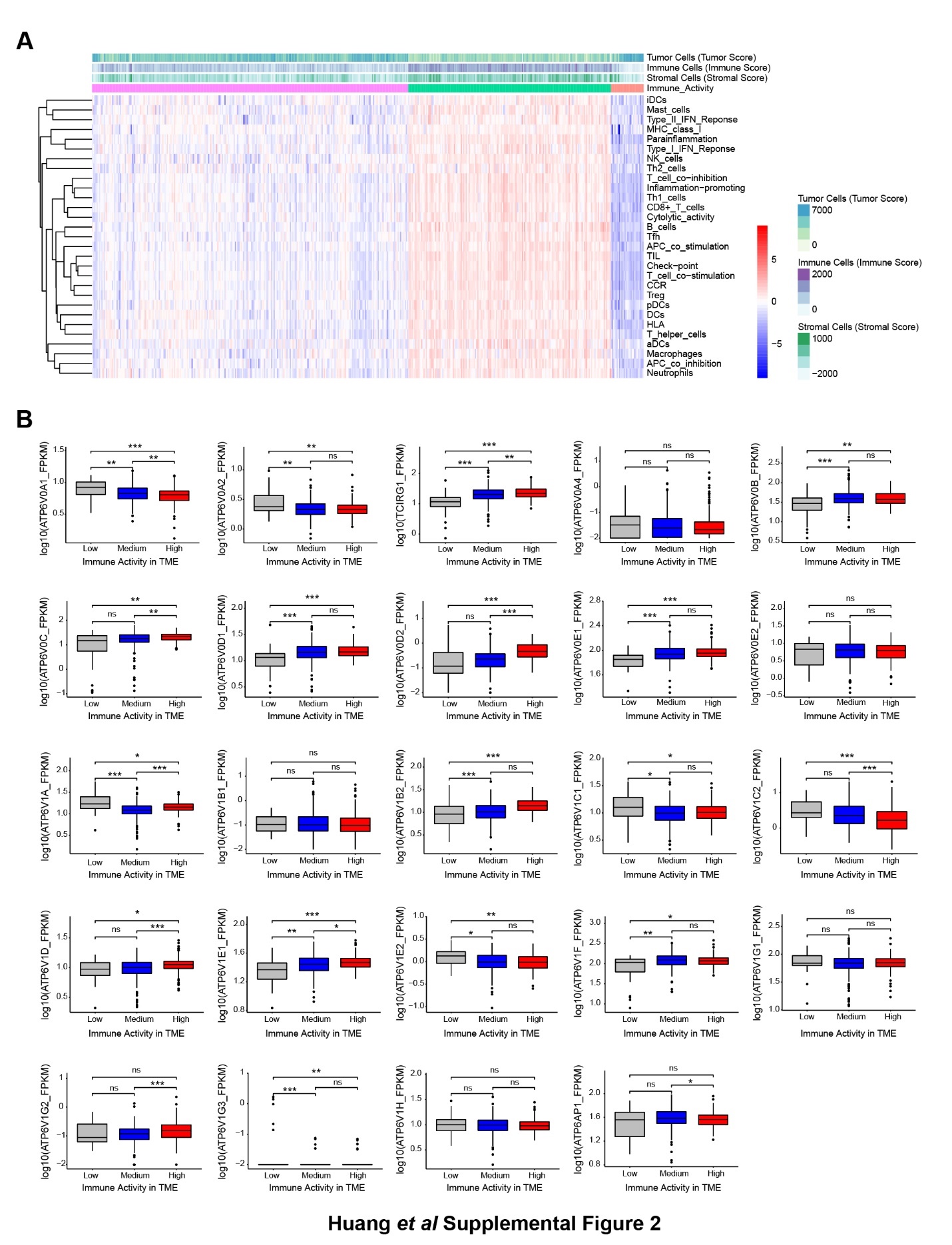


**Supplementary Fig. 2 ATP6V0A1 is inversely correlated with immune activity in human CRC.** **A**, The 471 TCGA-COAD samples were classified into three immune profile-defined clusters: CRC tissues with low (n=28), medium (n=270), and high (n=173) immune activity; their immune scores based on the transcriptomic levels of 29 distinct immune cells were shown in a heat map. **B**, The expression levels of various V-ATPase subunits were compared between the CRC samples with low, medium, or high immune activity, respectively. Statistical significance was determined using the Kruskal test.


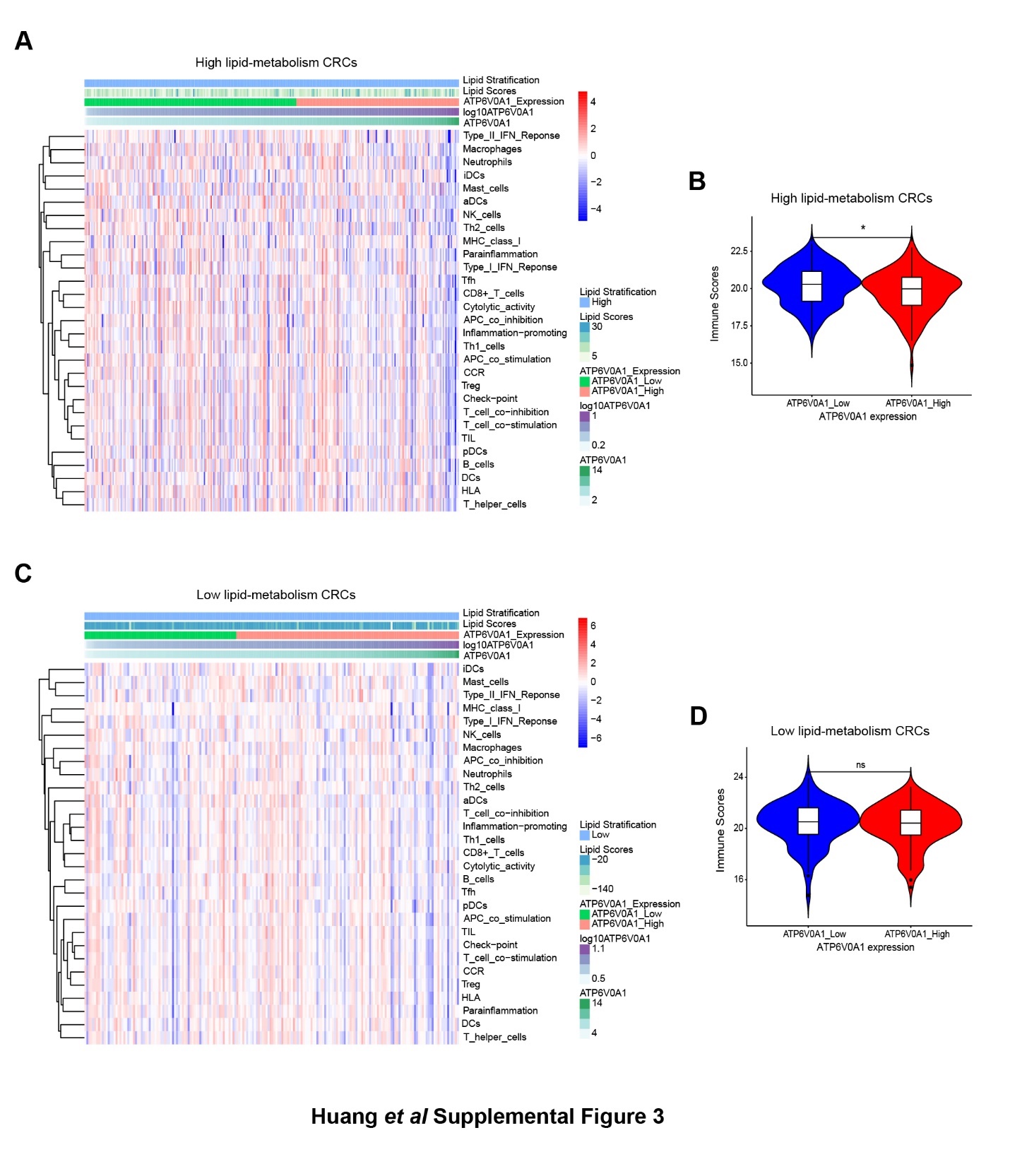


**Supplementary Fig. 3 High lipid metabolism is required to correlate A****TP6V0A1 expression and immune activity in CRC.** **A-B**, The correlation of ATP6V0A1 expression and immune activity was analyzed in CRCs with high lipid metabolism. A heat map showed the immune scores for the CRCs with high lipid metabolism, which were sorted in ascending order based on their ATP6V0A1 expression levels (**A**). Immune activity was compared between CRCs with low and high levels of ATP6V0A1 in relation to high lipid metabolism (**B**). n=158 (ATP6V0A1_Low) or 121 (ATP6V0A1_High) samples. **C-D**, The correlation of ATP6V0A1 expression and immune activity was analyzed in CRCs with low lipid metabolism. A heat map showed the immune scores for the CRCs with low lipid metabolism, which were sorted in ascending order based on their ATP6V0A1 expression levels (**C**). Immune activity was compared between CRCs with low and high levels of ATP6V0A1 in relation to low lipid metabolism (**D**). n=78 (ATP6V0A1_Low) or 114 (ATP6V0A1_High) samples. Statistical significance was determined using the Wilcoxon test.


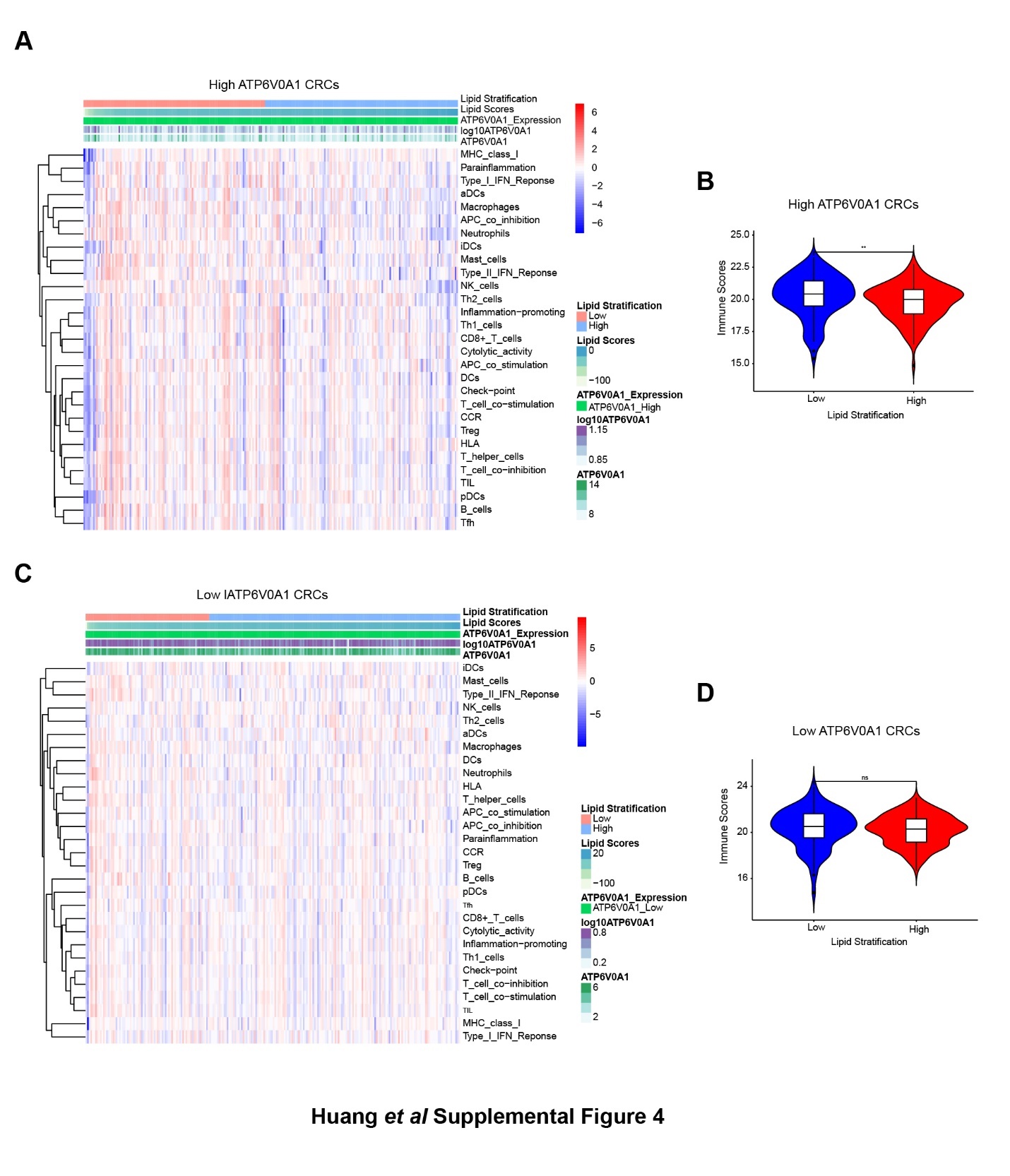


**Supplementary Fig. 4 ATP6V0A1 is essential for linking high lipid metabolism and low immune activity in CRC.** **A-B**, The correlation of lipid metabolism score and immune activity was analyzed in CRCs with high ATP6V0A1 expression. A heat map showed the immune scores for the CRCs with high ATP6V0A1 expression, which were sorted in ascending order based on their lipid metabolism score (**A**). Immune activity was compared between CRCs with low and high levels of lipid metabolism in relation to high ATP6V0A1 expression (**B**). n=114 (low lipid metabolism) or 121 (high lipid metabolism) samples. **C-D**, The correlation of lipid metabolism score and immune activity was analyzed in CRCs with low ATP6V0A1 expression. A heat map showed the immune scores for the CRCs with low ATP6V0A1 expression, which were sorted in ascending order based on their lipid metabolism score (**C**). Immune activity was compared between CRCs with low and high levels of lipid metabolism in relation to low ATP6V0A1 expression (**D**). n=78 (low lipid metabolism) or 158 (high lipid metabolism) samples. Statistical significance was determined using the Wilcoxon test.


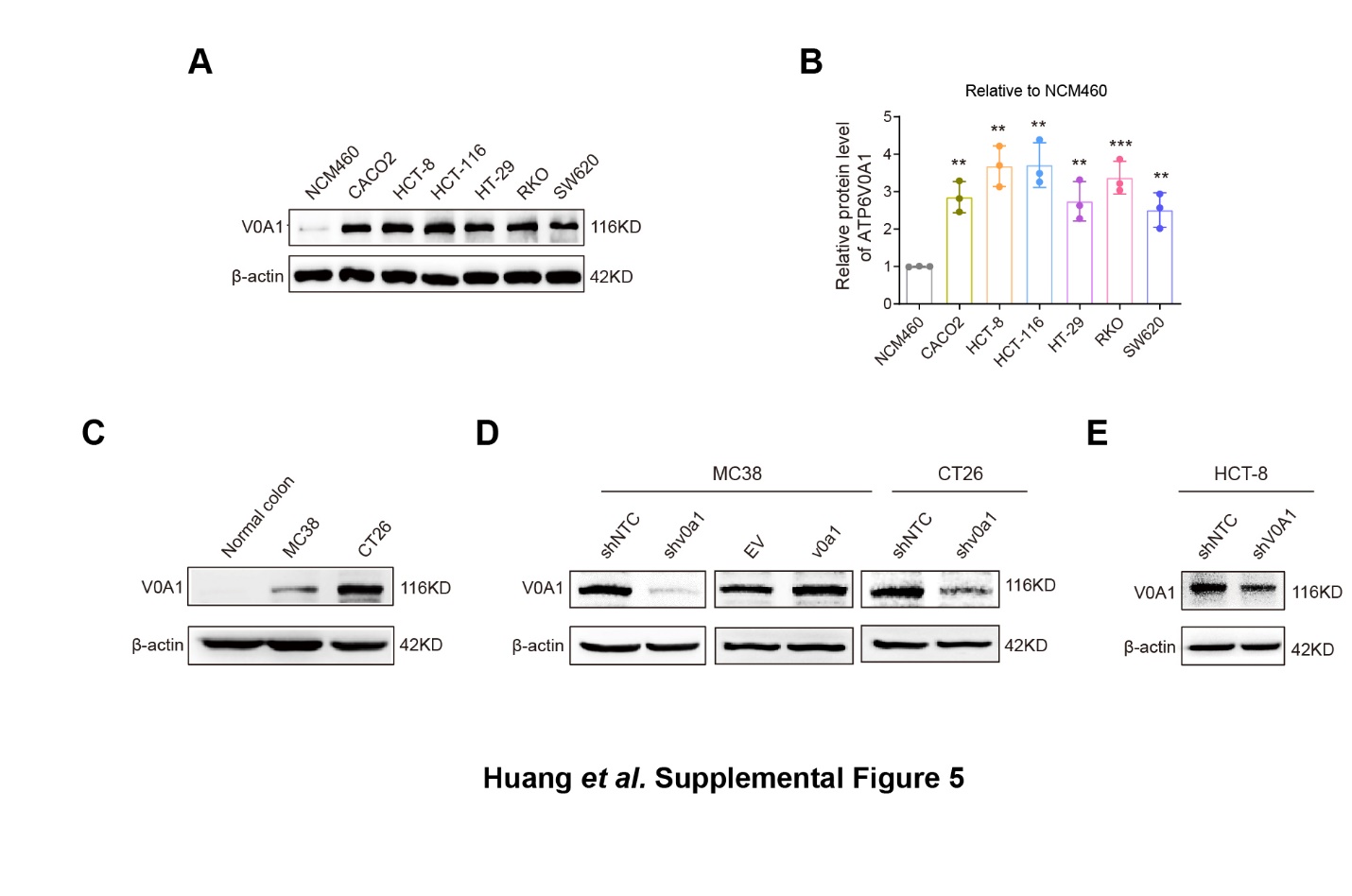


**Supplementary Fig. 5 ATP6V0A1 is frequently overexpressed in CRC cells.** **A-B**, The levels of ATP6V0A1 in different CRC cell lines were analyzed using western blotting. A representative image is shown in (**A**); the quantification of relative ATP6V0A1 protein level was analyzed based on three independent experiments by comparing the ATP6V0A1 protein level in CACO2, HCT-8, HCT-116, HT-29, RKO, or SW620 CRC cells with that in NCM460 normal colon cells (**B**). **C**, ATP6V0A1 protein levels in murine MC38 and CT26 CRC cells and normal colon epithelial cells were detected using western blotting. **D-E**, MC38, CT26, and HCT8 cells were used to generate *Atp6v0a1*/*ATP6V0A1* knockdown cells, and MC38 cells were used to construct *Atp6v0a1* over-expressing cells. For Supplementary Fig. 5**B**, data are shown as means  ±  s.e.m; **p < 0.01, ***p < 0.001; Statistical significance was determined between CACO2, HCT-8, HCT-116, HT-29, RKO, or SW620 CRC cells and NCM460 normal colon cells using unpaired two-sided Student’s t-test. Three independent experiments were performed for Supplementary Fig. 5**C**-**E**. Source data and exact p-value are provided as a Source Data file.


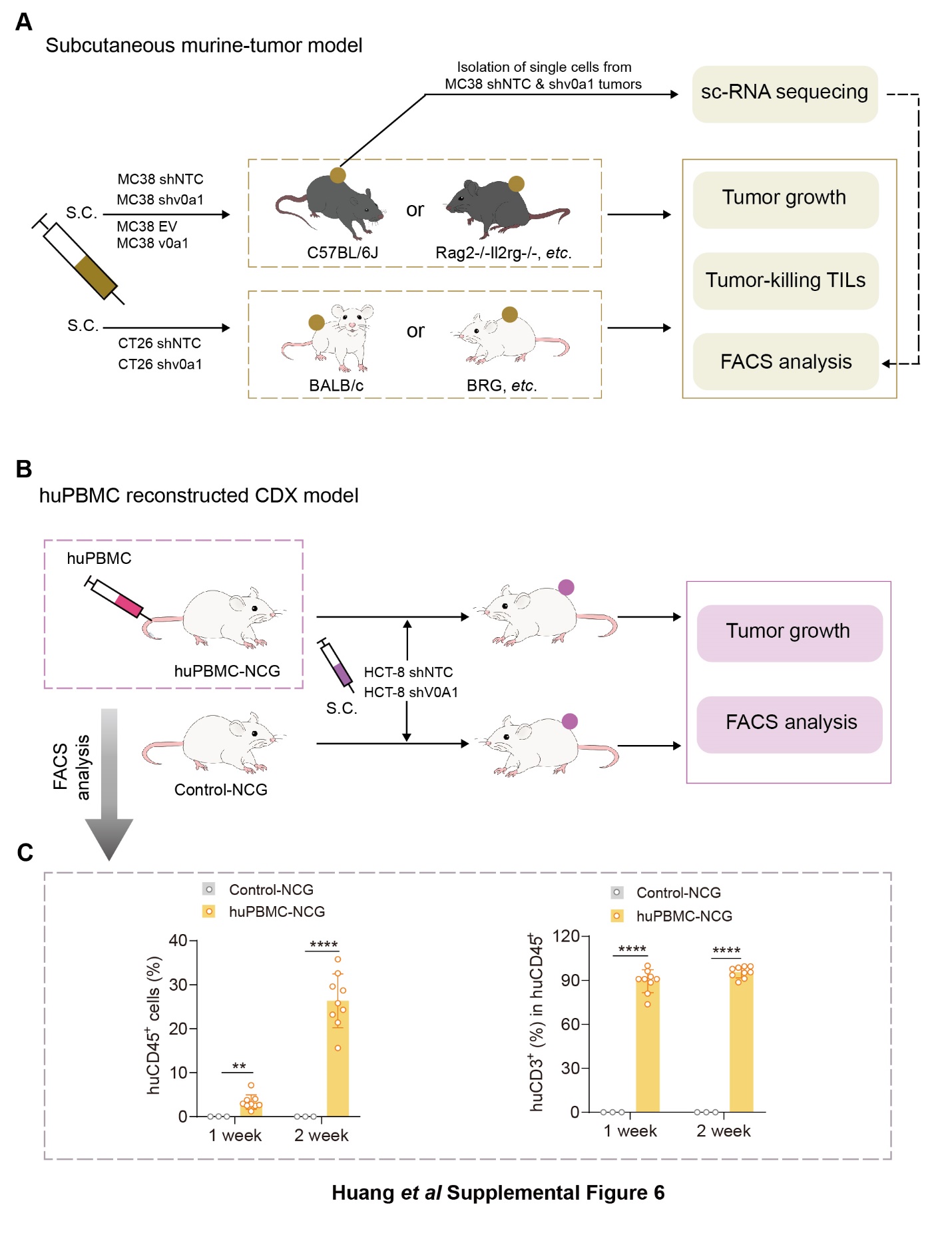
**Supplementary Fig. 6 Mouse and human CRC animal models used to assess the effect of tumor-derived ATP6V0A1 on antitumor immune responses.** **A,** MC38 cells transfected with the indicated plasmids were injected subcutaneously into immunocompetent C57/BL6 mice, immunodeficient Rag2^-/-^Il2rg^-/-^ or immunodeficient NOD/SCID mice, and CT26 cells transfected with the indicated plasmids were injected subcutaneously into immunocompetent BALB/c mice, immunodeficient BRG or immunodeficient NOD/SCID mice. For all tumor models, the tumor growth rate was assessed. For tumor models from immunocompetent mice, tumor tissue was also used for single-cell sequencing analysis, flow cytometry analysis, and the detection of the tumor-killing ability of TILs. **B,** Human peripheral blood mononuclear cells (PBMCs) were injected intravenously into immunodeficient NCG mice to construct immune-reconstituted huPBMC-NCG mice. For the cell line-derived xenograft (CDX), HCT-8-shNTC cells or HCT-8-shV0A1 cells were injected subcutaneously into immunoreconstituted huPBMC-NCG mice and immunodeficient NCG mice; tumor growth rates were assessed, and tumor tissues were used to analyze immunophenotypic changes by flow cytometry. **C,** Peripheral venous blood collected from the huPBMC-NCG mice in the first and second weeks following PBMC injection was analyzed for the proportion of human-derived CD45+ leukocytes and ratio of CD3+ T cells to CD45+ leukocytes by flow cytometry. The results showed that immunoreconstituted huPBMC-NCG mice were successfully constructed.


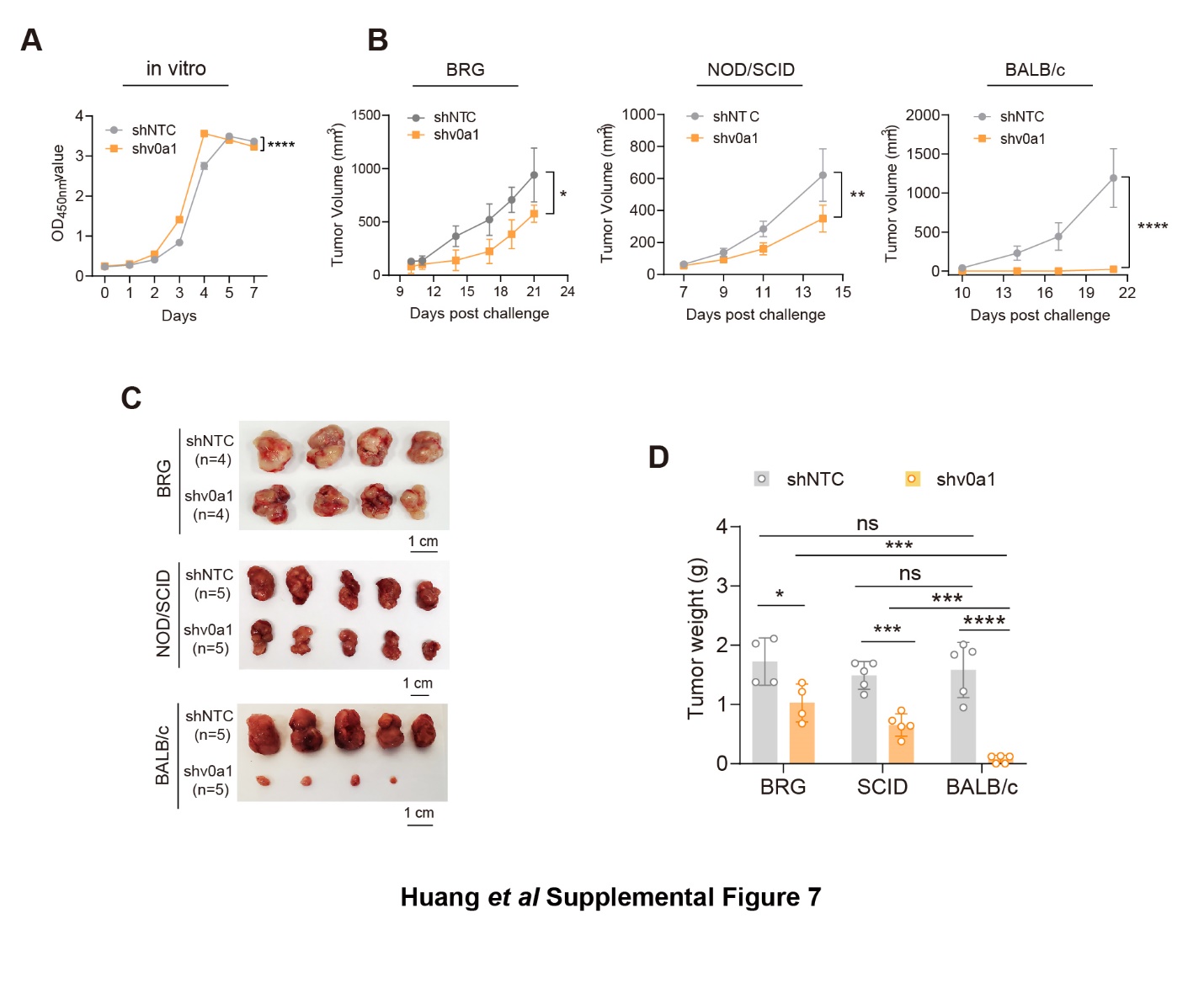


**Supplementary Fig. 7 The immune response is involved in the suppression of CT26 cell growth by ATP6V0A1 in vivo. A**, The growth of control CT26 (shNTC) cells and *Atp6v0a1*-knockdown (shv0a1) CT26 cells *in vitro* was analyzed by CCK8 assay. n=3 independent experiments. **B-D**, BRG mice, NOD/SCID mice, and BALB/c mice were subcutaneously injected with CT26 shNTC cells or CT26 shv0a1 cells as shown in Supplementary Fig. 6**A**. Tumor volumes were monitored using calipers, and average tumor growth curves were determined (**B**); photographs of the tumors are shown (**C**). Tumor weights at the termination time points are shown (**D**). n= 4 (BRG) or 5 (NOD/SCID and BALB/c) mice in each group. For all experiments, data are shown as means  ±  s.e.m; *p < 0.05, **p < 0.01, ***p < 0.001, ****p < 0.0001. Statistical significance was determined using ordinary two-way ANOVA in (**A**) and (**B**) and unpaired two-sided Student’s *t*-test in (**D**). Source data and exact p-value are provided as a Source Data file.


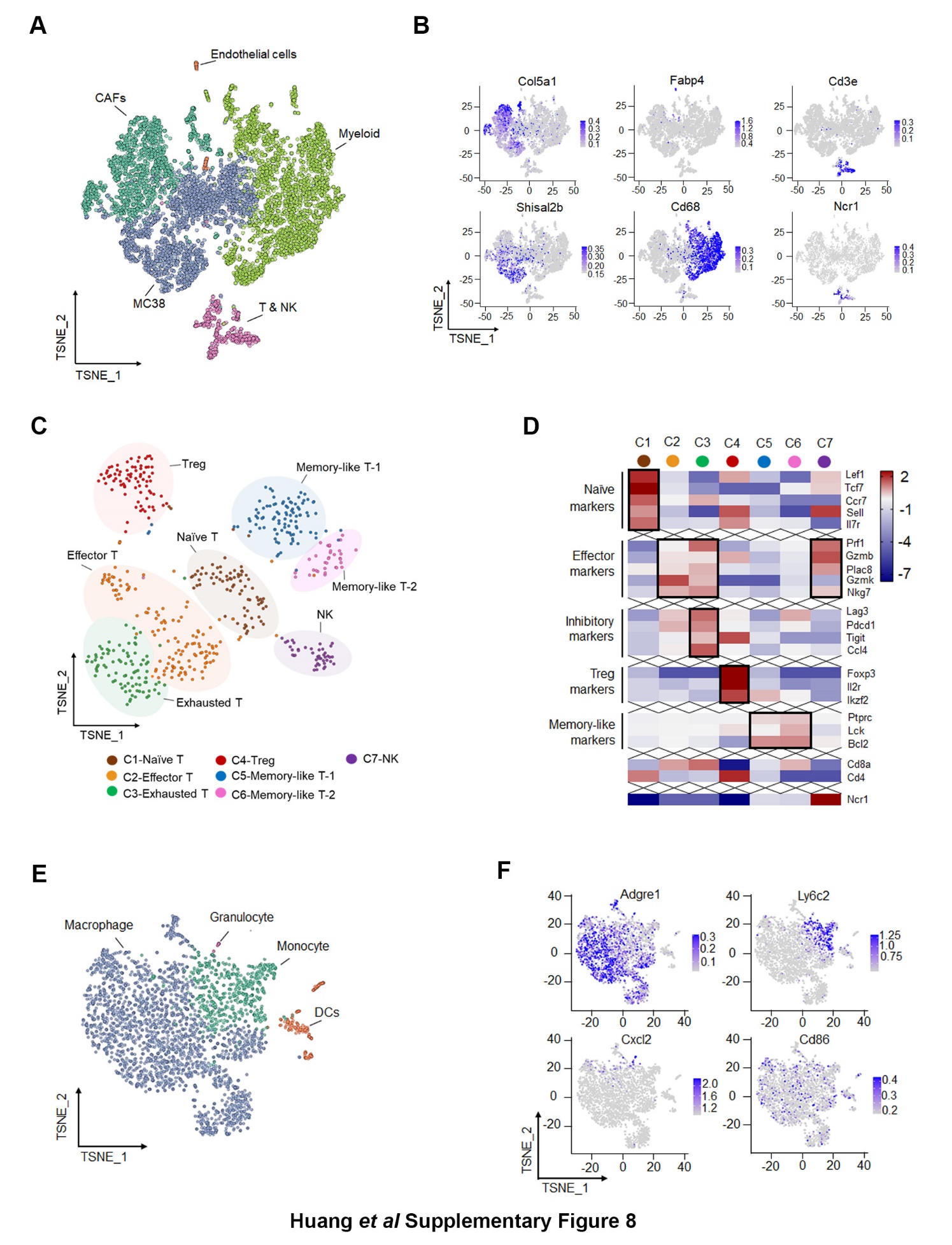


**Supplementary Fig. 8 scRNA-seq analysis reveals immune cell** **subpopulations.** Subcutaneous MC38-shNTC and MC38-shv0a1 tumors were isolated and disassociated into single cells for single cell-RNA seq analysis. **A-B**, Using the t-distributed stochastic neighbor embedding (t-SNE) method, cells from MC38-shNTC tumors and MC38-shv0a1 tumors were clustered into five subpopulations, including MC38 cells, CAFs, endothelial cells, myeloid cells, and T & natural killer (NK) cells (**A**); the expression of marker genes for each subpopulation is shown (**B**). **C-F**, Immune cells including T & natural killer (NK) cells and myeloid cells were further clustered into subpopulations using the t-distributed stochastic neighbor embedding (t-SNE) method. T and NK cells from MC38-shNTC and MC38-shv0a1 tumors were subclustered into seven subpopulations, including naïve T cells, effector T cells, exhausted T cells, Treg cells, NK cells, and two subtypes of memory-like T cells (**C**). The heatmap shows the expression levels of marker genes in each subpopulation (**D**). Myeloid cells from MC38-shNTC and MC38-shv0a1 tumors were further clustered into four subpopulations, including macrophages, granulocytes, monocytes, and dendritic cells (DCs) (**E**). The dot plots show marker gene expression levels for each cell-subpopulation (**F**).


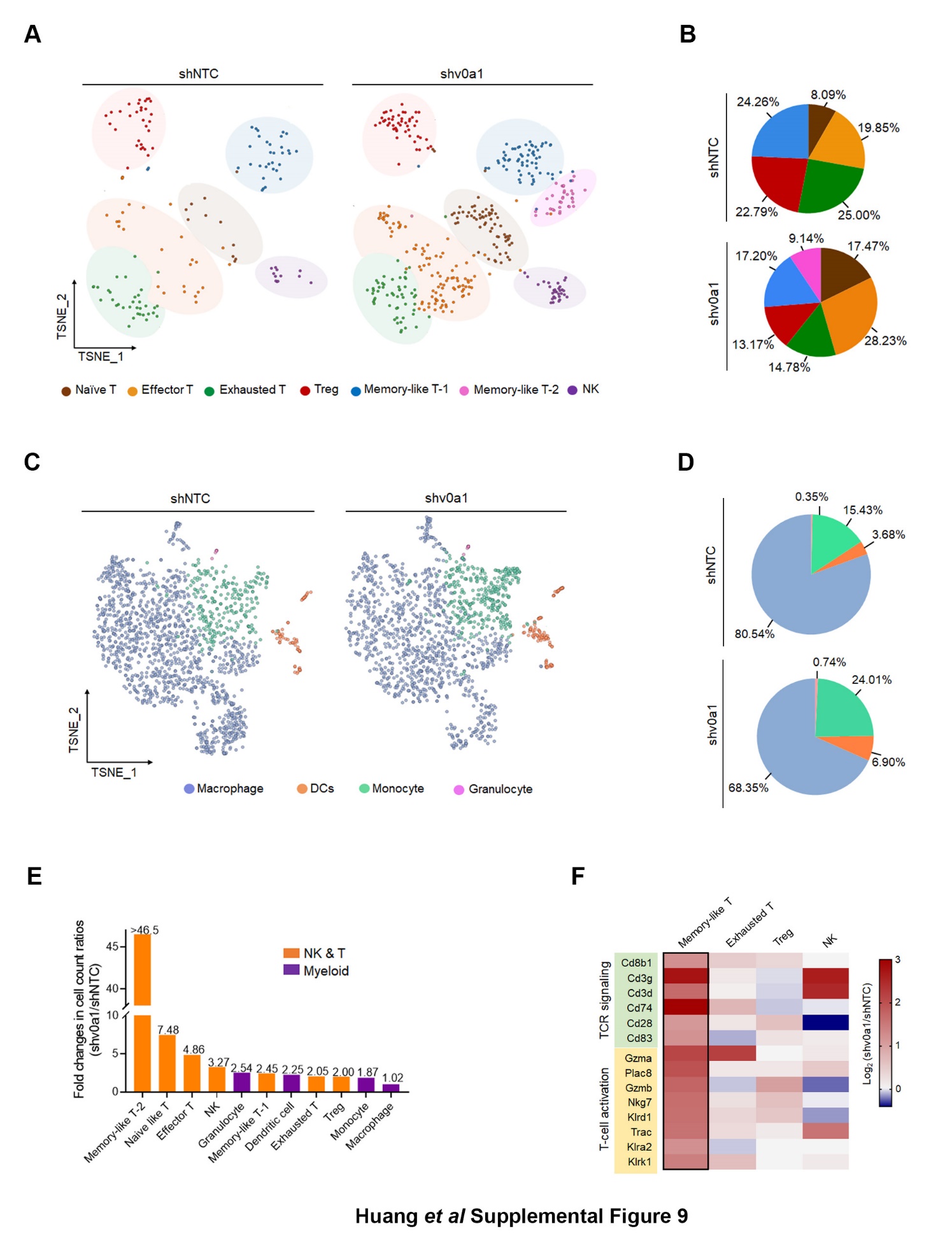


**Supplementary Fig. 9 Tumor-derived** **ATP6V0A1 rewires immune phenotypes in the TME.** Immune cell subpopulations from the scRNA-seq analysis of MC38 tumors (see Supplementary Fig. 8) were further analyzed for ATP6V0A1-regulated changes in immune phenotype. **A-B**, T and natural killer (NK) cell clusters were further clustered into seven subpopulations (**A**), which were analyzed for *Atp6v0a1*-interference mediated changes (**B**). **C-D**, Myeloid cell clusters were further clustered into four subpopulations (**C**), which were analyzed for *Atp6v0a1*-interference mediated changes (**D**). **E**, Comparison of fold changes in cell count ratios (shv0a1/shNTC) for the indicated immune cell subpopulations in MC38 tumors. **F**, Comparison of the relative expression levels of TCR signaling and T-cell activation genes in the indicated T-cell subpopulations in MC38-shNTC tumors and MC38-shv0a1 tumors.


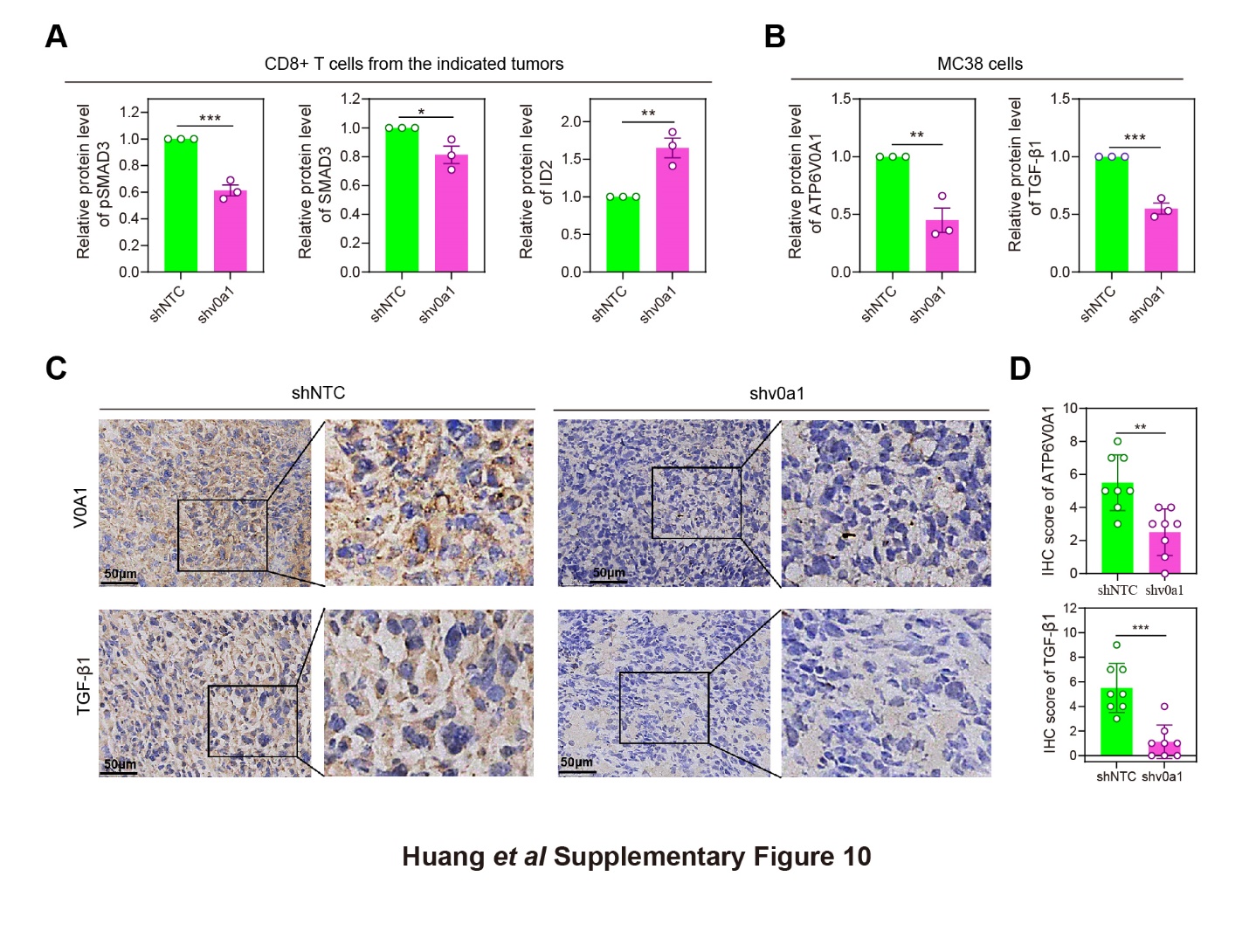


**Supplementary Fig. 10 ATP6V0A1 regulates paracrine TGF-β1/SMAD3 signaling in MC38 tumor. A**, Quantification analysis for the protein level of pSMAD3, SMAD3, and ID2 in CD8^+^ T cells detected by western blotting in Fig. 4B. n=3 independent experiments. **B**, The quantification for the western blotting data detecting MC38-derived ATP6V0A1 and TGF-β1 levels in Fig. 4E was analyzed. n=3 independent experiments. **C-D**, Atp6v0a1 interference suppresses TGF-β1 expression in MC38 tumors. Tissue sections from MC38-shNTC or MC38-shv0a1 tumors were analyzed for ATP6V0A1 and TGF-β1 expression using immunohistochemistry. Presentative images were shown (**C**); quantitative analysis of ATP6V0A1 or TGF-β1 expression were carried out (**D**; n=8 fields/group). Scale bar = 50 μm. Data representative of 3 independent experiments. For all experiments, data are shown as means ± s.e.m. *p < 0.05, **p < 0.01, ***p < 0.001. Statistical significance was determined using unpaired two-sided Student’s t-test. Source data and exact p-value are provided as a Source Data file.


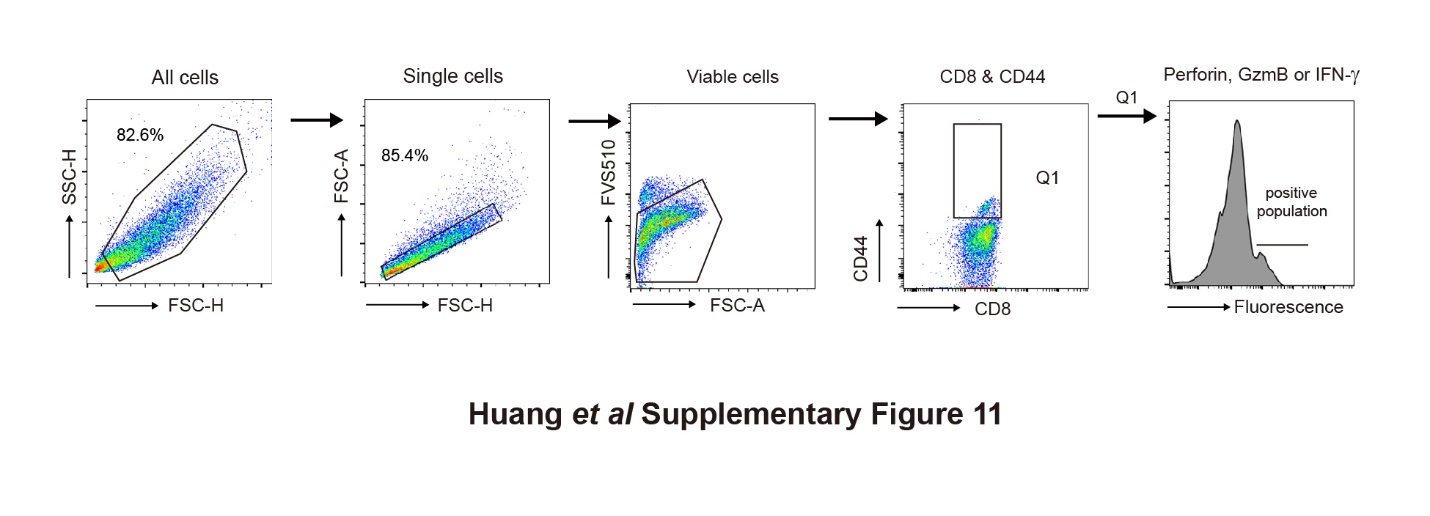


**Supplementary Fig. 11 Flow cytometry (FCM) strategy for gating viable CD44^+^CD8^+^ T cells and detecting effector cytokines.** The FCM strategy for gating viable CD44^+^CD8^+^ T cells and detecting effector cytokines in the indicated treated CD8^+^ T cells described in Fig. 4G was shown.


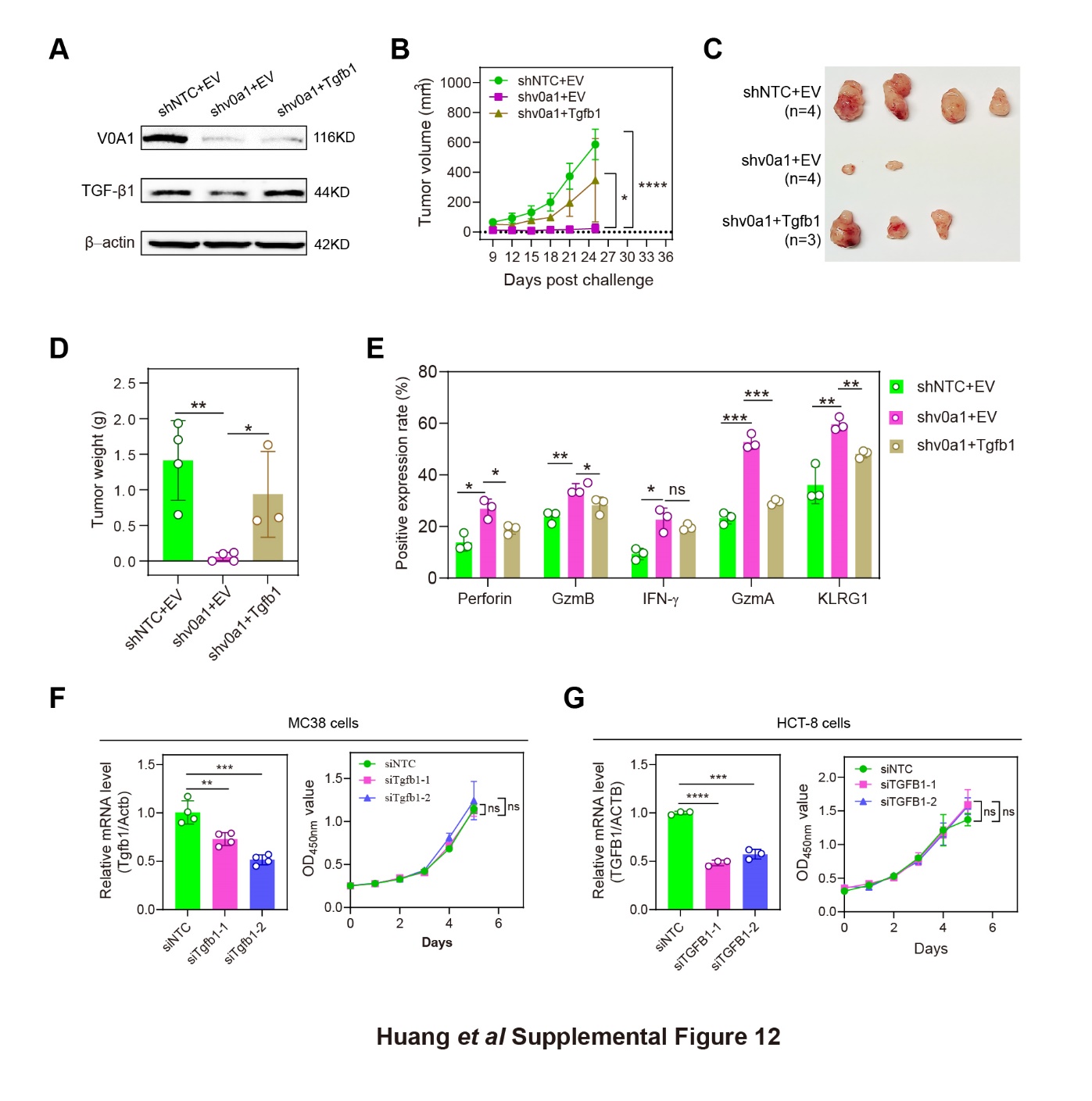


**Supplementary Fig. 12 Restoring *Tgfb1* attenuated the MC38 tumor suppression caused by *Atp6v0a1* depletion. A,** *Tgfb1* is expressed in *Atp6v0a1*-suppressing MC38 cells, and western blotting was used to detect the protein level of ATP6V0A1, TGF-β1, and β-actin. The samples derive from the same experiment but different gels for ATP6V0A1, β-actin, another for TGF-β1 were processed in parallel. **B**-**E**, the cells described in A were subcutaneously injected into C57BL/6J mice. n=4 (shNTC + EV and shv0a1 + EV) or 3 mice (shv0a1 + Tgfb1) in each group. Average tumor growth curves (**B**), photographs of the tumors (**C**), and a comparison of tumor weights on day 25 (**D**) are shown. TILs from day 25 tumors were analyzed for effector production in CD44^+^CD8^+^ T cells (**E**; n=3 mice per group). Data representative of three independent experiments. **F**-**G**, The effects of *Tgfb1/TGFB1* knockdown on the growth of MC38/HCT-8 cells were analyzed by CCK8 assay. n=4 independent experiments. For all experiments, data are shown as means ± s.e.m. *p < 0.05, **p < 0.01, ***p < 0.001, ****p < 0.0001. Statistical significance was determined using ordinary two-way ANOVA (in B, F, and G) or unpaired two-sided Student’s t-test (in D and E). Source data and exact p-value are provided as a Source Data file.


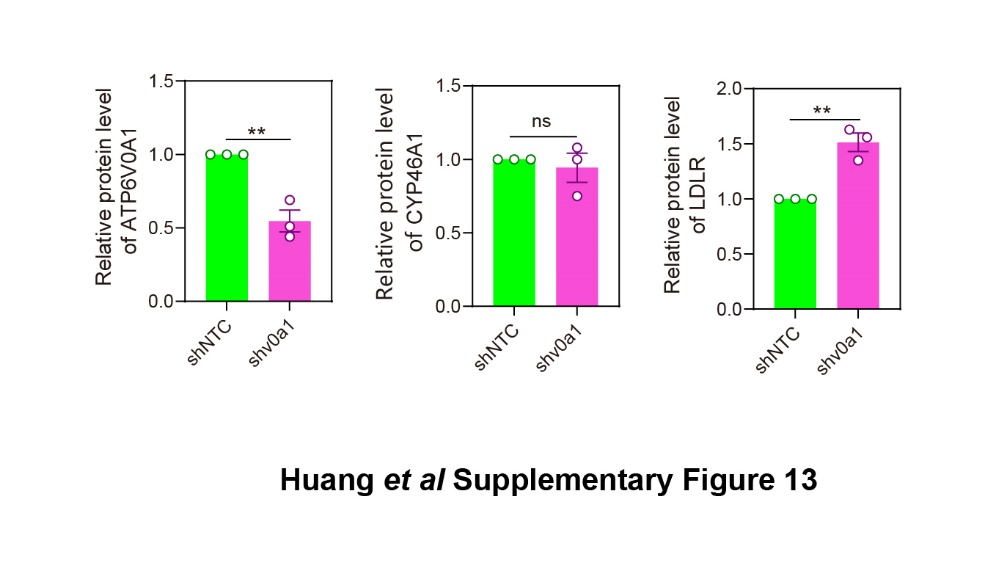


**Supplementary Fig. 13 Quantification analysis for the western blotting data in Fig. 5C.** The quantification was analyzed for the western blotting data in Fig. 5C based on three independent experiments. n=3 independent experiments. For all experiments, data are shown as means ± s.e.m. **p < 0.01. Statistical significance was determined using unpaired two-sided Student’s t-test. Source data and exact p-value are provided as a Source Data file.


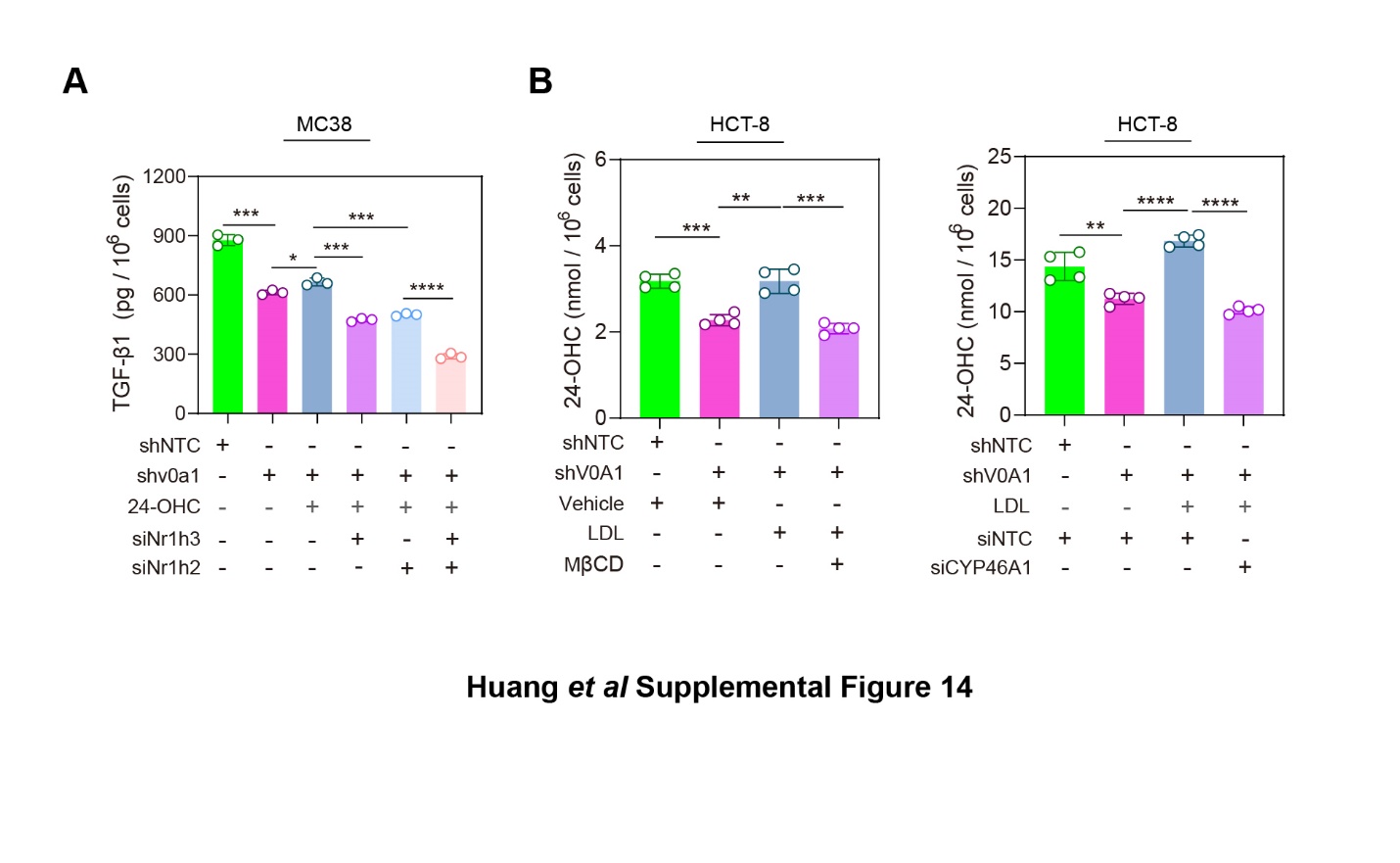


**Supplementary Fig. 14 Supplementary data relative to Fig. 5. A**, Both LXRα and LXRβ are essential for ATP6V0A1-induced TGF-β1 expression via 24-OHC production. MC38 (shNTC and shv0a1) cells were treated with 24-OHC in the absence or presence of Nr1h3- or Nr1h2-targeted siRNAs, and TGF-β1 levels in the supernatants were analyzed by ELISA. n=3 independent experiments. **B**, Cholesterol depletion by MβCD or 24-OHC suppression by CYP46A1 knockdown eliminates the roles of exogenous LDL restoring TGF-β1 expression reduced by ATP6V0A1 depletion. HCT-8 (shNTC and shV0A1) cells were treated with LDL in the absence or presence of MβCD (**B**, left) or with LDL in the presence of siNTC or siCYP46A1 (**B**, right); ELISA detected 24-OHC levels in the supernatant. n=4 independent experiments. For all experiments, data are shown as means ± s.e.m; *p < 0.05, **p < 0.01, ***p < 0.001, ****p < 0.0001. Statistical significance was determined using unpaired two-sided Student’s t-test. Source data and exact p-value are provided as a Source Data file.


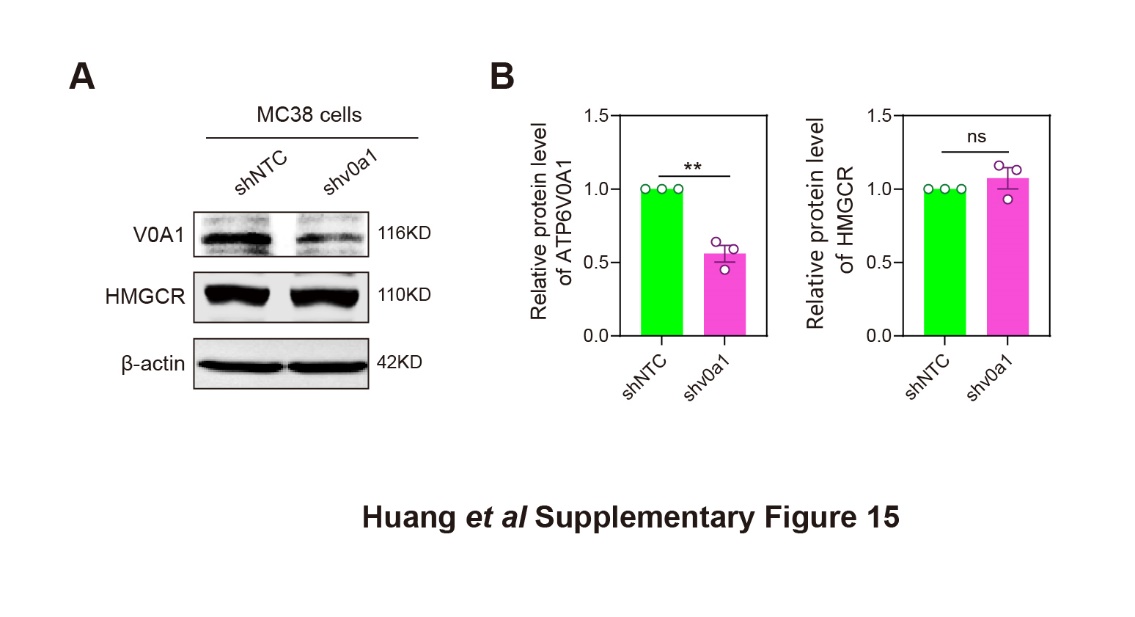


**Supplementary Fig. 15 *Atp6v0a1* knockdown does not change HMGCR expression.** Western blotting was used to detect HMGCR protein levels in MC38-shNTC and MC38-shv0a1 cells. Representative blots were shown (**A**). Three independent experiments were performed, and the quantification of protein expression was analyzed (**B**). For Supplementary Fig. 15**A**, the samples derive from the same experiment but different gels for ATP6V0A1, β-actin, another for HMGCR were processed in parallel. For Supplementary Fig. 15**B**, data are shown as means ± s.e.m. **p < 0.01. Statistical significance was determined using unpaired two-sided Student’s t-test. Source data and exact p-value are provided as a Source Data file.


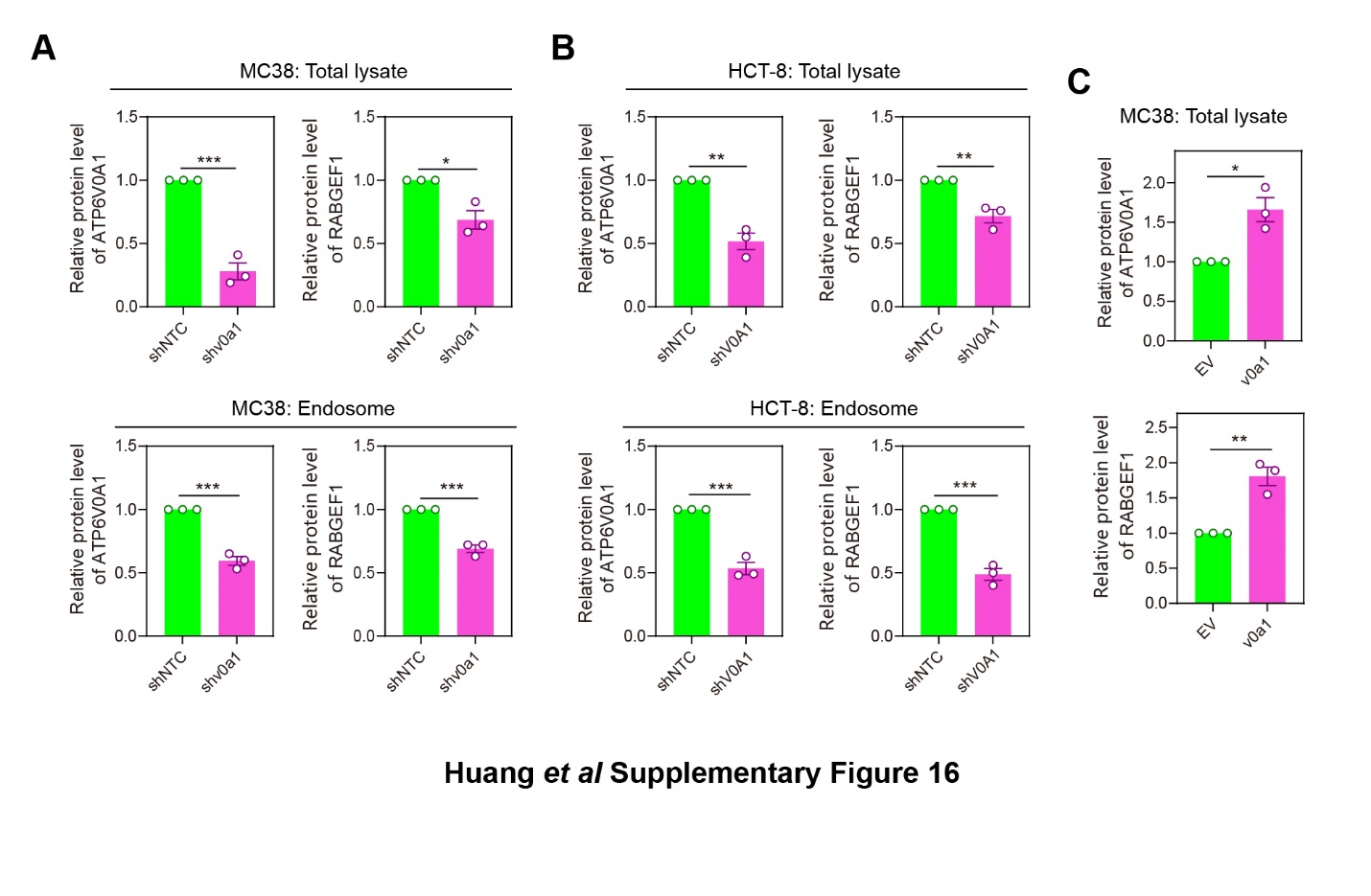


**Supplementary Fig. 16 Quantification analysis for the western blotting data in Fig. 6. A-C**, Quantification analysis for the western blotting data in Fig. 6B (**A**), 6C (**B**), and 6D (**C**). n=3 independent experiments. For all experiments, data are shown as means ± s.e.m. *p < 0.05, **p < 0.01, ***p < 0.001. Statistical significance was determined using Student’s t-test. Source data and exact p-value are provided as a Source Data file.


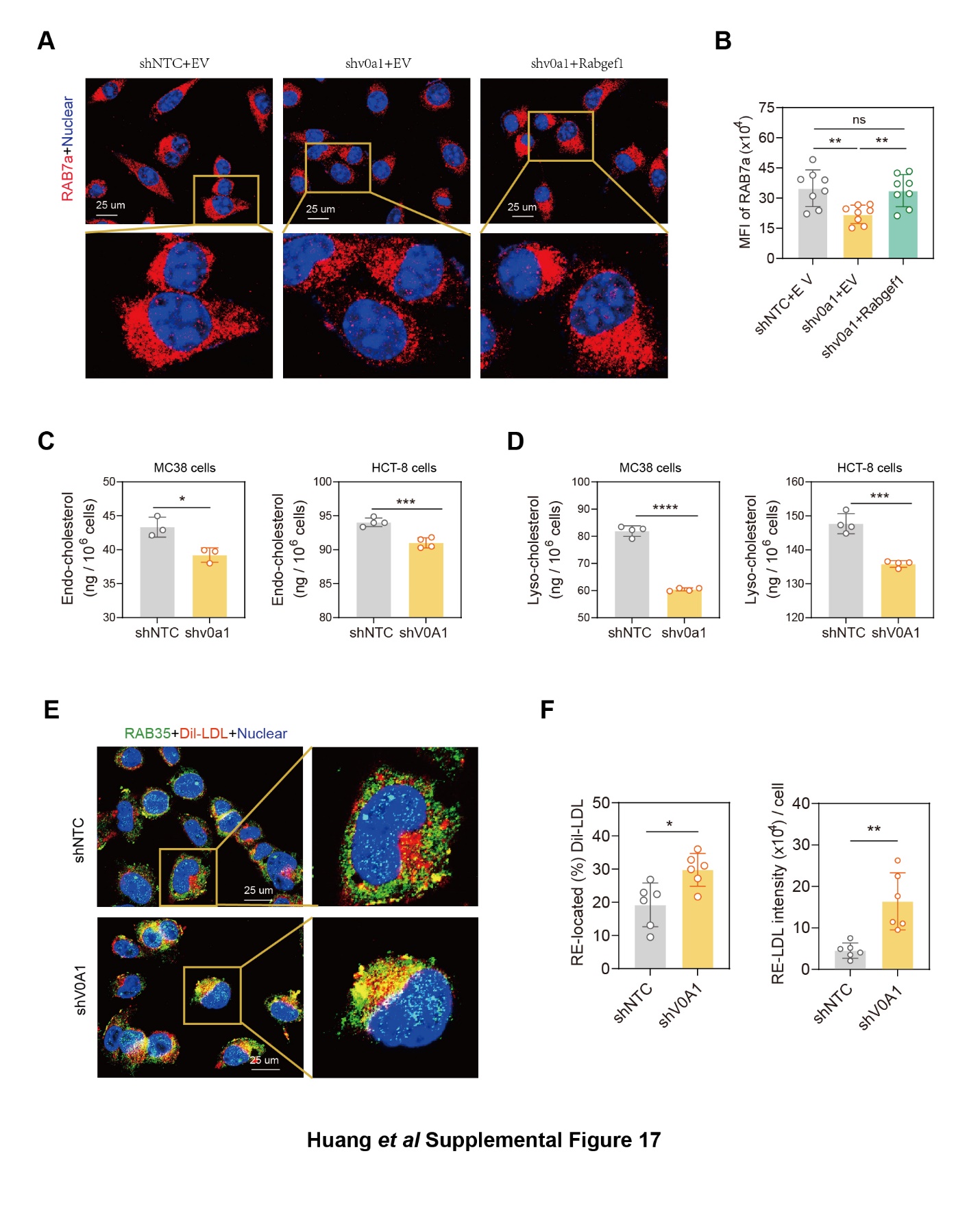


**Supplementary Fig. 17 ATP6V0A1 supports RABGEF1-dependent endosome maturation and alters cholesterol levels in transport vesicles.** **A-B**, The levels of RAB7a (red) in cellular vesicles were evaluated using confocal fluorescence microscopy in the indicated cells. Representative images are shown in (**A**), and the mean fluorescence intensity of vesicle-derived RAB7a (fluorescence intensity/cell) was analyzed based on eight fields of images in each group, using Image J software (**B**; n=8 fields/group). **C-D**, Endosomes (**C**) and lysosomes (**D**) were isolated from MC38 or HCT-8 cells, and their lipids were extracted and analyzed for cholesterol levels using the Amplex™ Red cholesterol assay. n=3 (C, left) or 4 (C, right; D) independent experiments. **E-F**, HCT-8 cells were treated with 50 µg/ml of Dil-labeled human LDL for 6 hours, and confocal fluorescence microscopy was used to analyze the colocalization of Dil-LDL with RAB35+ recycling endosomes. Representative images (**E**) and quantitative analysis are shown; the percentage of Dil-LDL located in RAB35^+^ recycling endosomes (RE) and mean fluorescence intensity of RE-located Dil-LDL were determined using Image J software (**F**; n=6 fields/group). For all experiments, data are shown as means ± s.e.m; *p < 0.05, **p < 0.01, ***p < 0.001, ****p < 0.0001. Statistical significance was determined using unpaired two-sided Student’s t-test. Three independent experiments were performed for Supplementary Fig. 17**A** and **E**. Source data and exact p-value are provided as a Source Data file.


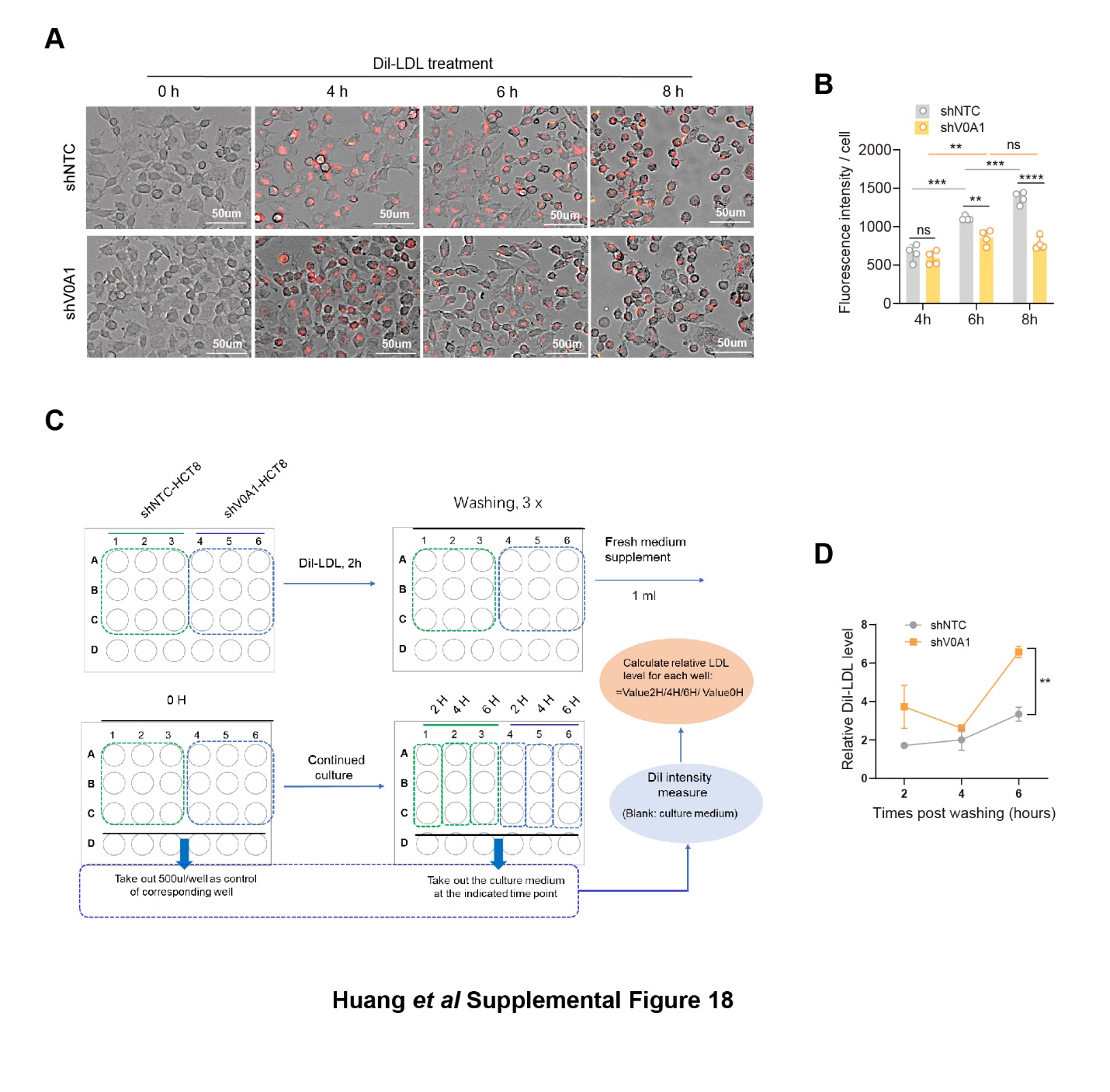


**Supplementary Fig. 18 *ATP6V0A1* knockdown modulates the transport of exogenous LDL in HCT-8 cells. A-B**, HCT-8 shNTC or HCT-8 shV0A1 cells were treated with 50 µg/ml of Dil-labeled human LDL for the indicated times, and fluorescence microscopy was carried out to analyze the levels of exogenous cholesterol in cells. Representative images (**A**) and quantitative analysis (**B**) are shown; for each group, intracellular Dil fluorescence intensity was quantified in the cells from four intact fields at 10x magnification using Image J software (**B**). Data representative of 3 independent experiments. **C-D**, The strategy to determine the level of exogenous LDL recycling to culture medium is shown in (**C**); at different time points during continuous culture, HCT-8 cell supernatants were analyzed for Dil-LDL recycling via the measurement of Dil intensity using a fluorescence microplate reader (**D**; n=3 independent experiments). For all experiments, data are shown as means  ±  s.e.m; *p < 0.05, **p < 0.01, ***p < 0.001, ****p < 0.0001. Statistical significance was determined using ordinary two-way ANOVA in (**D**) and unpaired two-sided Student’s *t*-test in (**B**). Source data and exact p-value are provided as a Source Data file.


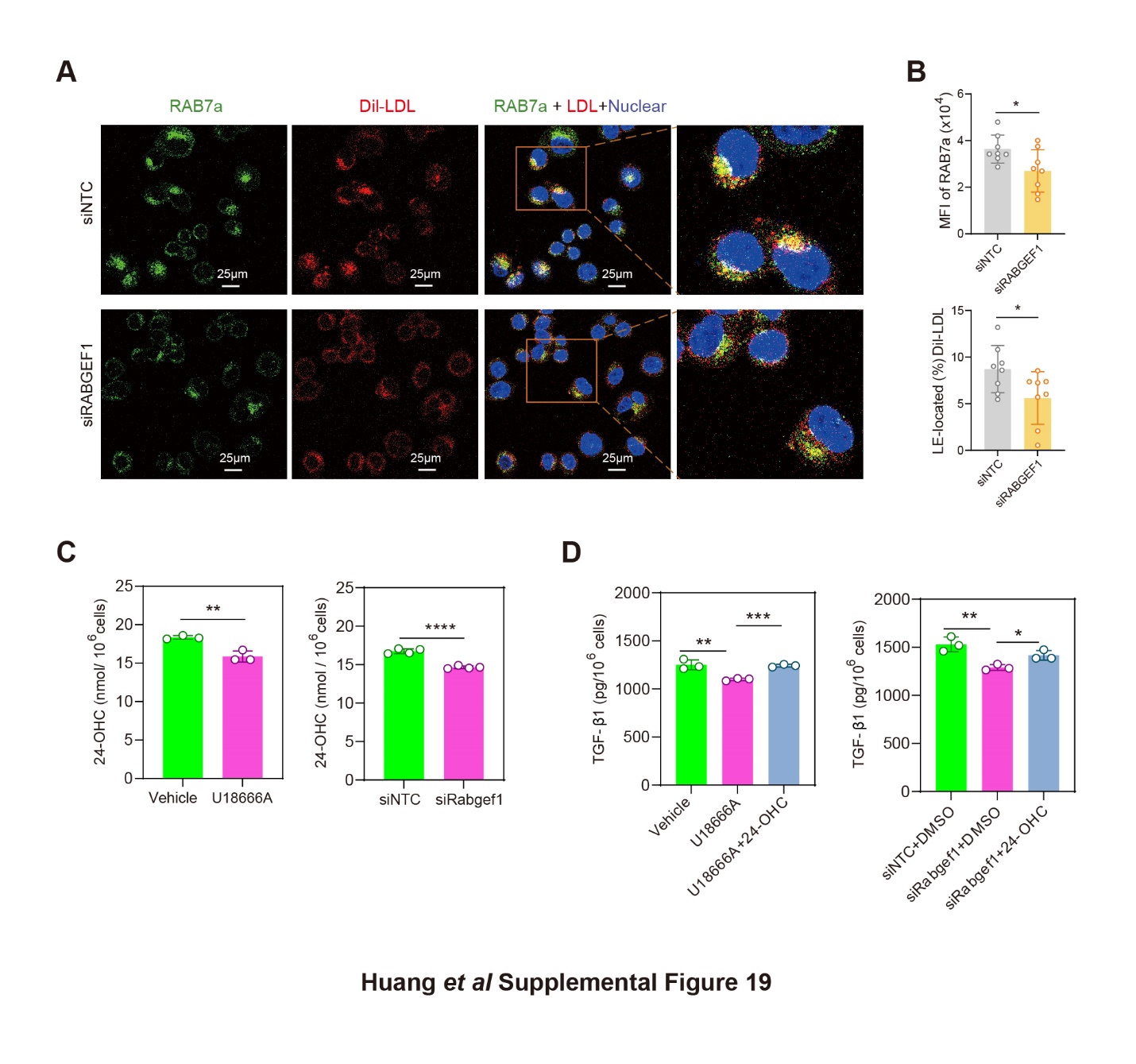


**Supplementary Fig. 19 RABGEF1 is required for the absorption of exogenous LDL, which supports TGF-β1 expression.** Control (siNTC) or *RABGEF1* knockdown (siRABGEF1) HCT-8 cells were treated with 50ug/ml of Dil-LDL for 6 hours, and then incubated with antibodies for RAB7a labeling. RAB7a (Green) and Dil-LDL (red) in these cells were detected using confocal fluorescence microscopy (**A**); quantitative analysis of vesicle-derived RAB7a levels (**B**, upper) and the percentages of Dil-LDL that were located in RAB7a+ late endosome (LE; **B**, lower) was carried out using Image J. n=8 fields/group; Data representative of 3 independent experiments. **C-D**, HCT-8 cells were treated as the indication, and the level of 24-OHC (**C**) and TGF-β1 (**D**) in the supernatant was detected by ELISA. n=3 (C, left; D) or 4 (C, right) independent experiments. For all experiments, data are shown as means  ±  s.e.m; *p < 0.05, **p < 0.01, ***p < 0.001, ****p < 0.0001. Statistical significance was determined using unpaired two-sided Student’s *t*-test. Source data and exact p-value are provided as a Source Data file.


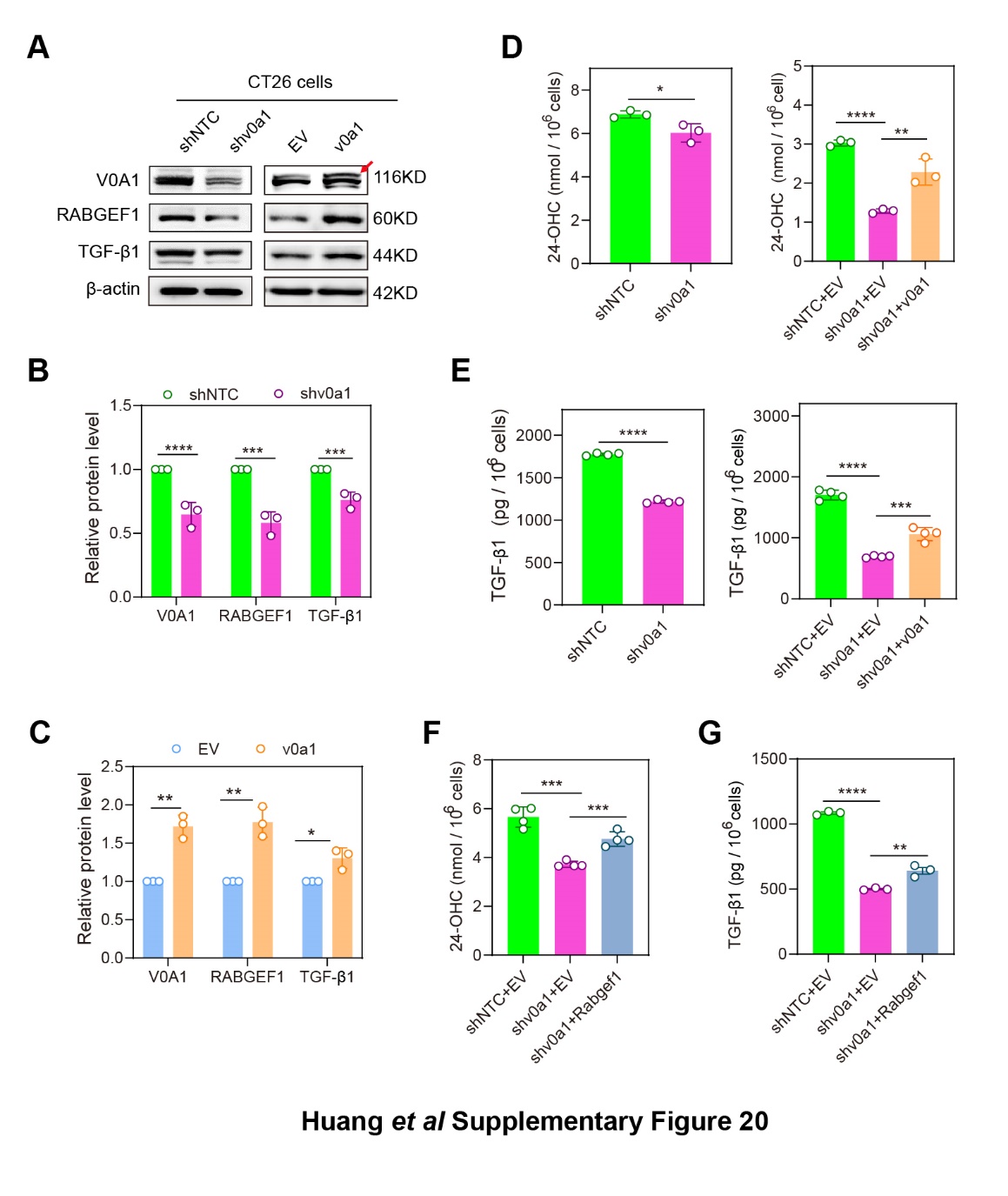


**Supplementary Fig. 20 ATP6V0A1 promotes 24-OHC production and TGF-β1 expression via RABGEF1 pathway in CT26 cell model. A-C**, Atp6v0a1 was knocked down or overexpressed in CT26 cells, and western blotting was used to detect the protein level of ATP6V0A1, RABGEF1, TGF-β1, and β-actin. Representative blots were shown; The red arrow points to the assumed ATP6V0A1 protein band (**A**). The quantification of protein expression was analyzed based on three independent experiments (**B-C**). **D-G**, *Atp6v0a1* (**D**-**E**)*,* or *Rabgef1* (**F**-**G**) was expressed in *ATP6v0a1*-suppressing CT26 cells; 24-OHC (**D, F**) and TGF-β1 (**E, G**) in the supernatant were analyzed by ELISA. n=3 (**D** and **G**) and 4 (**E** and **F**) independent experiments. For all experiments, data are shown as means ± s.e.m; *p < 0.05, **p < 0.01, ***p < 0.001, ****p < 0.0001. Statistical significance was determined using unpaired two-sided Student’s *t*-test. Source data and exact p-value are provided as a Source Data file.


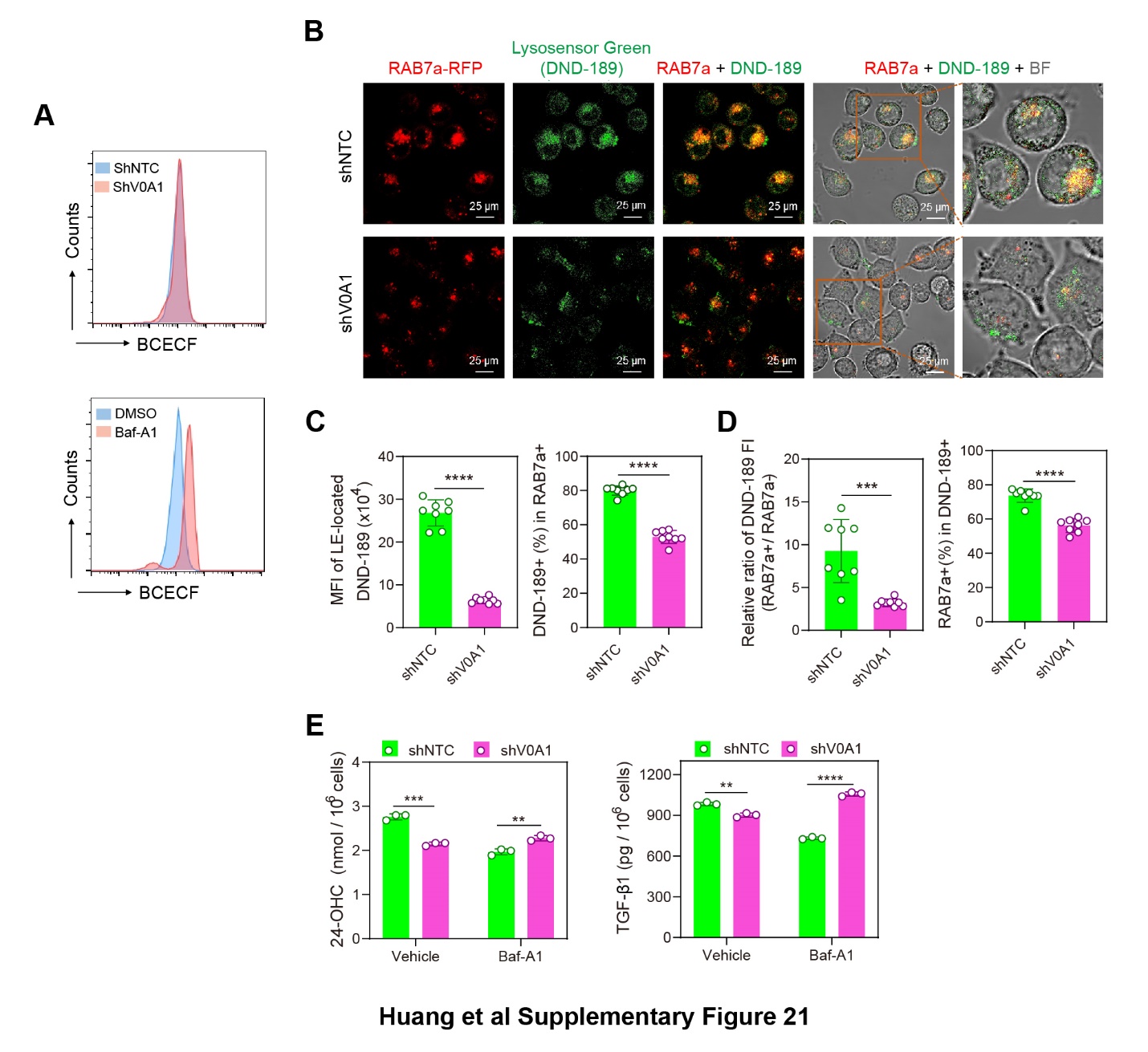


**Supplementary Fig. 21 The roles of V-ATPase acidification-activity in ATP6V0A1-regulated TGF-β1 expression.** **A**, BCECF-AM, a pH probe, was incubated with ATP6V0A1-suppressing HCT-8 cells or with wild type HCT-8 cells treated with Baf-A1; The fluorescence-intensity of BCECF in cells was measured by FACS to assess the cellular pH. Data representative of 3 independent experiments. **B**-**D**, The HCT-8 cells transfected with CellLight™ RAB7a-RFP (BacMam 2.0) were incubated with Lysosensor Green DND-189, and confocal fluorescence microscopy was used to analyze the fluorescence-intensity of Lysosensor Green DND-189 in RAB7a^+^ late endosomes. Representative images are shown in (**B**). Quantitative analyses of DND-189 localization in RAB7a^+^ endosomes were carried out by measuring the fluorescence intensity (**C**, left) or the ratio (**C**, right) of DND-189 located in these vesicles with Image J. Image J was used to compare the pH changes between RAB7a^+^ late endosomes and other vesicles by analyzing the ratio of DND-189 intensity in RAB7a^+^ late endosomes to that in other vesicles (**D**, left) or analyzing the percentage of RAB7a^+^DND-189^+^ late endosomes in all DND-189^+^ vesicles (**D**, right). n=8 fields/group; Data representative of 3 independent experiments. **E**, ATP6V0A1-suppressing HCT-8 cells were treated with vehicle or Baf-A1, and 24-OHC or TGF-β1 in the supernatant was measured by ELISA. n=3 independent experiments. For all experiments, data are shown as means ± s.e.m; **p < 0.01, ***p < 0.001, ****p < 0.0001. Statistical significance was determined using unpaired two-sided Student’s t-test. MFI, mean fluorescence intensity= fluorescence intensity/cell. Source data and exact p-value are provided as a Source Data file.


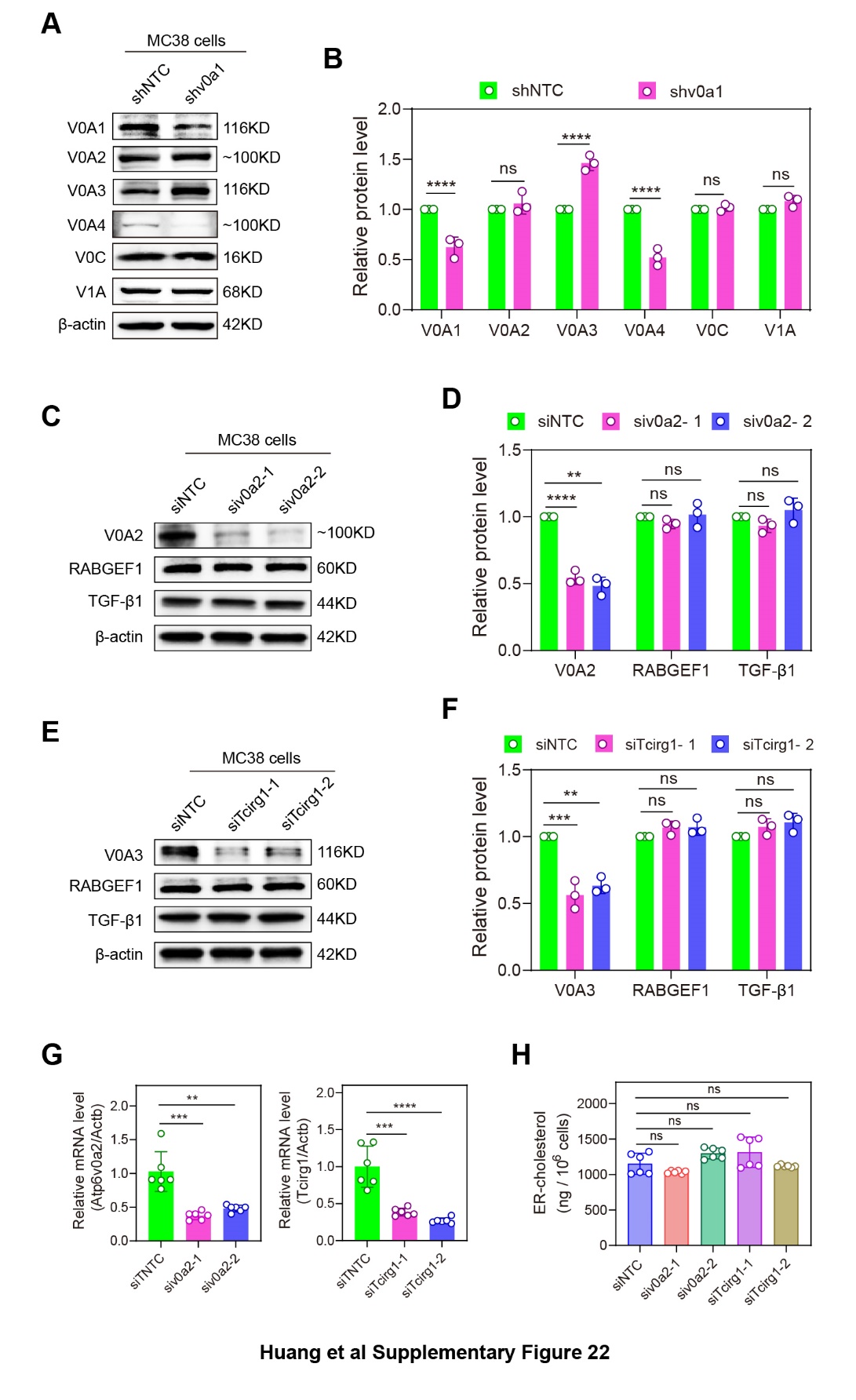


**Supplementary Fig. 22 The expression alteration of other V0A subunits induced by ATP6V0A1 suppression and their effects on the expression of RABGEF1 and TGF-β1. A-B**, Western blotting was used to detect ATP6V0A1, ATP6V0A2, ATP6V0A3, ATP6V0A4, ATP6V0C and ATP6V1A protein levels in MC38-shNTC and MC38-shv0a1 cells. Representative blots were shown (**A**), and the quantification of protein expression was analyzed based on three independent experiments (**B**). **C-F**, *Atp6v0a2* or *Tcirg1* (translated to ATP6V0A3 protein) was knocked down in MC38 cells, and western blotting detected the protein levels of RABGEF1, TGF-β1, and β-actin. Representative blots were shown (**C**, **E**), and the quantification of protein expression was analyzed based on three independent experiments (**D**, **F**). **G-H**, MC38 cells were treated with Atp6v0a2- or Tcirg1-targeted siRNAs. The knockdown efficiency of Atp6v0a2 or Tcirg1 was assessed by q-PCR (**G**). Following the ER isolation and lipid extraction from the indicated cells, cholesterol levels were detected using the Amplex™ Red cholesterol assay (**H**). n=6 replicates; Data pooled from 3 independent experiments. For Supplementary Fig. 22**A**, the samples derive from the same experiment but different gels for ATP6V0A1, ATP6V0C, ATP6V1A, β-actin, another for ATP6V0A2, another for ATP6V0A3 and another for ATP6V0A4 were processed in parallel. For Supplementary Fig. 22**A**, the samples derive from the same experiment but different gels for ATP6V0A1, ATP6V0C, ATP6V1A, β-actin, another for ATP6V0A2, another for ATP6V0A3 and another for ATP6V0A4 were processed in parallel. For Supplementary Fig. 22**C** and **E**, the samples derive from the same experiment but different gels for ATP6V0A2/ATP6V0A3, RABGEF1, β-actin, another for TGF-β1 were processed in parallel. For all experiments, data are shown as means ± s.e.m; **p < 0.01, ***p < 0.001, ****p < 0.0001. Statistical significance was determined using unpaired two-sided Student’s t-test. Source data and exact p-value are provided as a Source Data file.


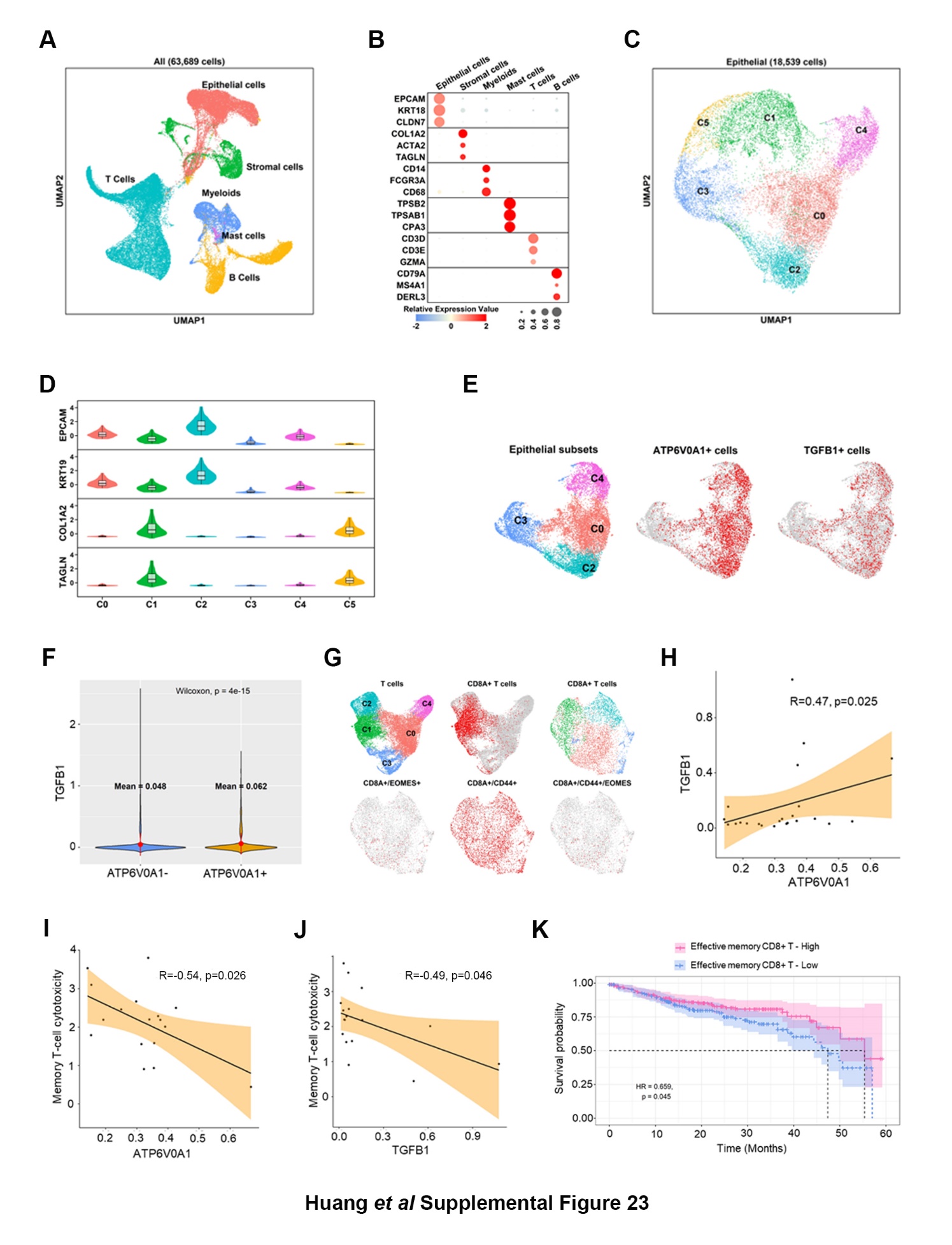


**Supplementary Fig. 23 ATP6V0A1 is positively correlated with TGFB1 and inversely correlated with memory CD8^+^ T cell effectiveness in clinical CRC samples. A-J**, Raw scRNA-seq data (GSE132465) for tumor tissues from 23 CRC patients were downloaded as described in Methods and analyzed for the correlations between tumor-derived *ATP6V0A**1*, tumor-derived *TGFB1*, and the effectiveness of memory CD8^+^ T cells. The UMAP method was used to cluster 63,689 cells into six subpopulations (**A**), annotated as epithelial cells, stromal cells, mast cells, T cells, and B cells using the markers shown in (**B**). Epithelial cells were further clustered into six subpopulations (**C**), which were evaluated for their expression of epithelial and stromal marker genes (**D**). Epithelial cell subpopulations with high expression of *EPCAM* and *KRT19* and low expression of *COL1A2* and *TAGLN* were selected for the analysis of *ATP6V0A1* and *TGFB1* expression (**E**). The selected epithelial cells were divided into groups with/without *ATP6V0A1* expression and analyzed for *TGFB1* expression (**F**). CD8^+^ T cells were selected from the T cell subpopulations and analyzed for the expression of memory cell markers, including *CD44* and *EOMES* (**G**). Tumor samples (n=17) with more than two memory CD8^+^ T cells were chosen for the analysis of correlations between tumor-derived *ATP6V0A1*, tumor-derived *TGFB1*, and the effectiveness of memory CD8^+^ T cells (**H-J**). **K**, Effective memory CD8+ T cells identified by a signature of CD8, EOMES, CD44, GZMA, PRF1, and KLRG1 were analyzed for their correlation with patient overall survival based on TCGA-COAD database.


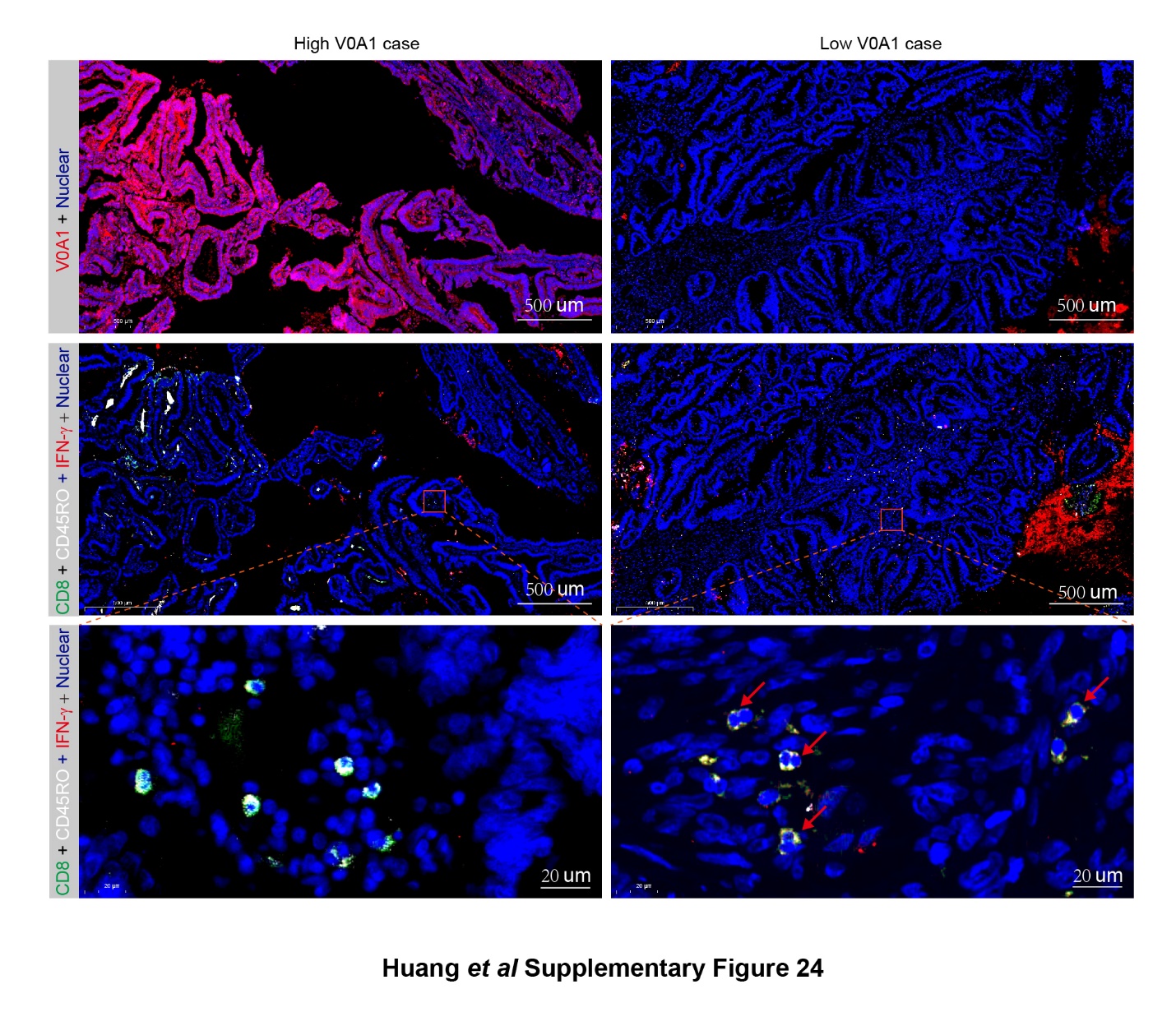


**Supplementary Fig. 24 Detection of ATP6V0A1 and CD45RO^+^CD8^+^ T-cell effectiveness in human CRC.** Paraffin-embedded tumor sections from 32 CRC patients were stained with antibodies against ATP6V0A1 and IFN-γ^+^CD45RO^+^CD8^+^ T cells. Representative immunofluorescence (IF) images for ATP6V0A1 and CD45RO^+^CD8^+^ T-cell effectiveness (IFN-γ expression rate) between high- and low-ATP6V0A1 cases were shown.


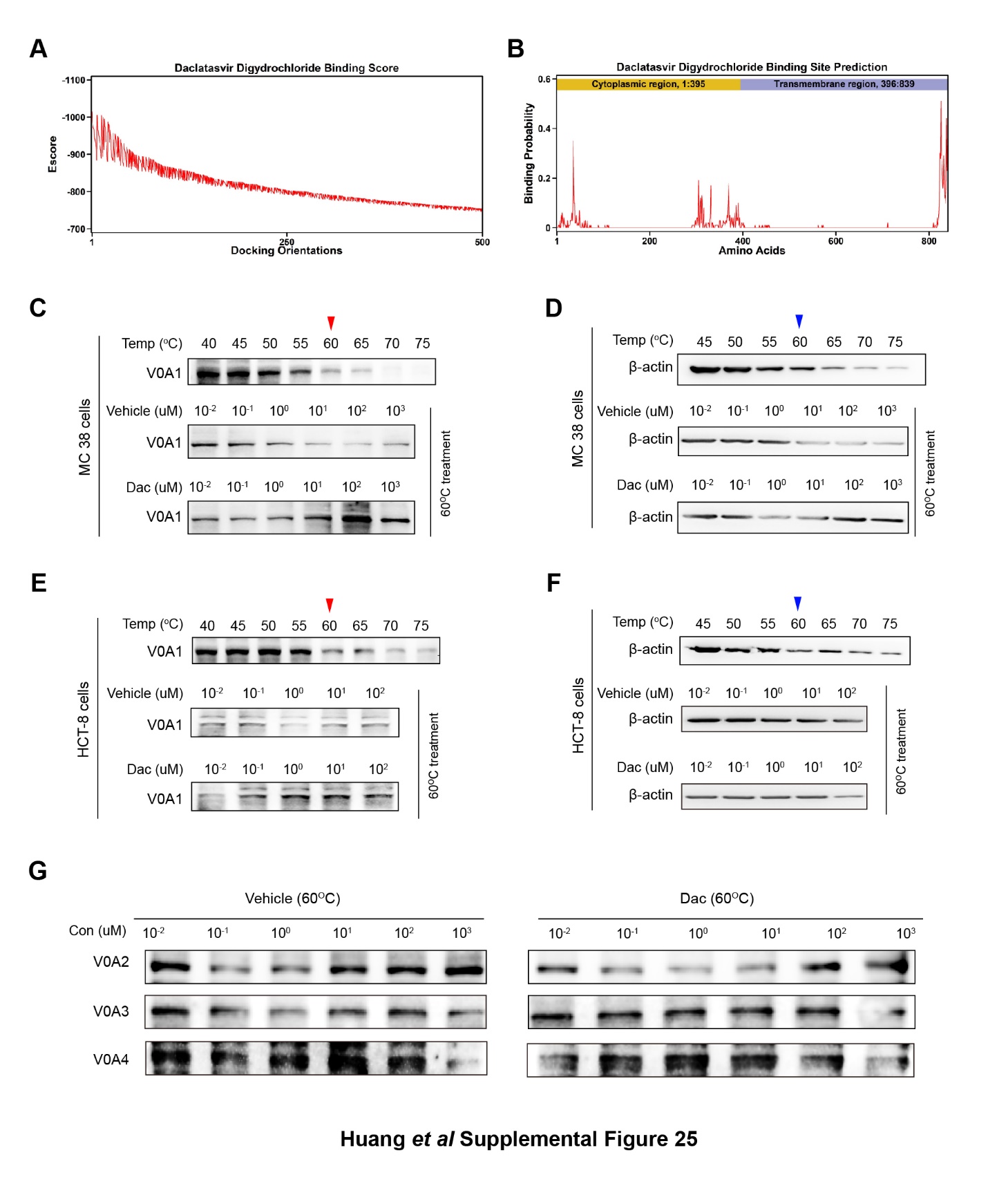


**Supplementary Fig. 25 Dac is able to bind to CRC tumor cell-intrinsic ATP6V0A1. A-B**, Using the molecular docking method, Daclatasvir (Dac), an FDA-approved small molecule compound, was predicted to be able to bind to ATP6V0A1 protein. The predicted binding score of Dac with murine ATP6V0A1 protein is shown in (**A**); the Dac binding site in murine ATP6V0A1 protein was also predicted (**B**). **C-F**, Cellular thermal shift assays were used to evaluate the binding of Dac to ATP6V0A1 protein in MC38 cells (**C**, **D**) and HCT-8 cells (**E**, **F**). MC38 cell lysates were heated to different temperatures, and precipitated proteins were removed by centrifugation; western blotting was then used to detect ATP6V0A1 (**C**, upper) and β-Actin (**D**, upper) in the soluble protein samples. Temperatures of 60°C and above significantly induced denaturation of ATP6V0A1 and β-Actin. MC38 cell lysates were also incubated with different concentrations of Dac or equal amounts of vehicle and heated to 60°C; western blotting was used to evaluate changes in the melting temperatures of ATP6V0A1 (**C**, lower) and β-Actin (**D**, lower). Similar experiments were performed in HCT-8 cells (**E**, **F**). **G**, Cellular thermal shift assays were used to evaluate the binding of Dac in MC38 cells to other V0A subtypes of V-ATPase, including ATP6V0A2, ATP6V0A3 (TCIRG1), and ATP6V0A4. MC38 cell lysates incubated with vehicle or Dac were heated to 60°C, and precipitated proteins were removed by centrifugation; western blotting was then used to detect ATP6V0A2, ATP6V0A3, and ATP6V0A4 in the soluble protein samples. The samples derive from the same experiment but different gels for ATP6V0A2, another for ATP6V0A3 and another for ATP6V0A4 were processed in parallel. Three independent experiments were performed for Supplementary Fig. 22C-G. Source data are provided as a Source Data file.


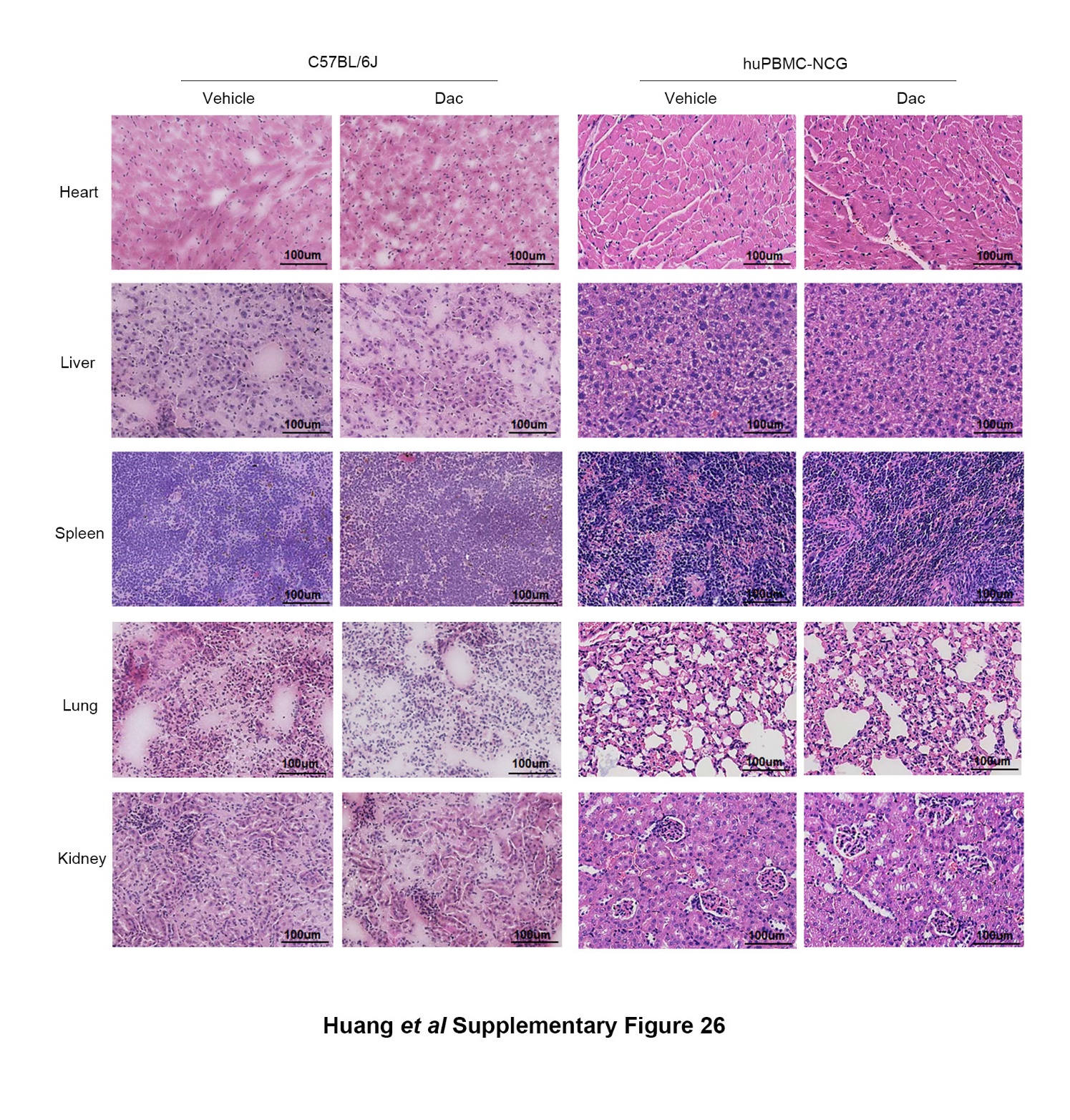


**Supplementary Fig. 26 Dac treatment does not induce organ toxicity in mice.** Following Dac treatment of MC38 tumors in C57BL/6J mice and HCT-8 tumors in huPBMC-NCG mice (Fig. 9), major organs (heart, liver, spleen, lung, and kidney) were isolated after the mice were sacrificed; the tissues were then embedded for sectioning and H&E staining. The H&E images show that no organic toxicity was induced by Dac treatment. Scale bar = 100μm. Data representative of three independent experiments.

**SUPPLEMENTARY TABLES**

Supplementary Table 1 provides Pearson’s Chi-squared analysis for the correlation of ATP6V0A1 and TGFB1 expression in CRCs.

Supplementary Table 2 provides the EOMES+CD44+CD8+ T cell counts for the CRC samples, which were analyzed in Supplementary Figure 23.

Supplementary Table 3 provides information on siRNA and shRNA sequences.

**Supplementary Table 1. Cell counts in the indicated cell subpopulations**

A scRNA-seq dataset (GSE132465) from the GEO database was used to analyze the expression of ATP6V0A1 and TGFB1 in CRC cells (see Supplementary Figure 23). The cell counts of ATP6V0A1+TGFB1+, ATP6V0A1+TGFB1-, ATP6V0A1-TGFB1+, and ATP6V0A1-TGFB1- subpopulations were shown, and the two-sided Pearson’s Chi-squared analysis was used to evaluate the correlation of ATP6V0A1 and TGFB1 expression.

| **Group** | **ATP6V0A1+** | **ATP6V0A1-** | **Total** |
| --- | --- | --- | --- |
| **TGFB1+** | 460 | 930 | 1390 |
| **TGFB1-** | 2803 | 9316 | 13049 |
| **Total** | 3263 | 10246 | 14439 |
| X-squared = 67.042; df = 1; p value = 2.658e-16 | | | |

**Supplementary Table 2.** **EOMES+CD44+CD8+ T cell counts in individual tumor samples**

The EOMES+CD44+CD8+ T cell counts for the CRC samples analyzed in Supplementary Figure 23 were shown.

| **Sample** | SMC06-T | SMC05-T | SMC07-T | SMC01-T | SMC11-T | SMC08-T | SMC10-T | SMC14-T |
| --- | --- | --- | --- | --- | --- | --- | --- | --- |
| **Cell counts** | 32 | 30 | 22 | 18 | 12 | 11 | 10 | 9 |
| **Sample** | SMC02-T | SMC24-T | SMC04-T | SMC15-T | SMC19-T | SMC03-T | SMC22-T | SMC17-T |
| **Cell counts** | 8 | 8 | 7 | 7 | 7 | 5 | 5 | 4 |
| **Sample** | SMC25-T | SMC09-T | SMC21-T | SMC20-T | SMC23-T | SMC16-T | SMC18-T |  |
| **Cell counts** | 3 | 2 | 2 | 1 | 1 | 0 | 0 |  |

**Supplementary Table 3.** **siRNA sequences and shRNA sequences targeting different genes**

| **Name** | **Targeted gene** | **Sequence** |
| --- | --- | --- |
| siNTC | nontarget | Purchased from Guangzhou RiboBio Co., Ltd.  Catalogue number: siN0000001-1-5 siR NC #1 |
| siv0a2-1 | murine *Atp6v0a2* | Sense:5'-GAGAAGUCACCAAGAACAAGG-3';  Anti-sense:5'-UUGUUCUUGGUGACUUCUCUG-3' |
| siv0a2-2 | murine *Atp6v0a2* | Sense:5'-AGAAGAUAUGUGACUGUUACC-3';  Anti-sense:5'-UAACAGUCACAUAUCUUCUUA-3' |
| siTcirg1-1 | murine *Tcirg1* | Target: 5'-CGGACTGCTCATGTTTCTCTT-3' |
| siTcirg1-2 | murine *Tcirg1* | Target: 5'-GAGTTCAGAGACCTCAACGAA-3' |
| siRabgef1 | murine *Rabgef1* | Purchased from Guangzhou RiboBio Co., Ltd.  Catalogue number: siB14218111646-1-5 |
| siRABGEF1 | human *RABGEF1* | Purchased from Guangzhou RiboBio Co., Ltd.  Catalogue number: SIGS0001726-1 |
| siCYP46A1 | human *CYP46A1* | Purchased from Guangzhou RiboBio Co., Ltd.  Catalogue number: SIGS0001450-1 |
| shNTC | nontarget | 5'-CCTAAGGTTAAGTCGCCCTCT-3' |
| shv0a1-1 | murine *Atp6v0a1* | 5'-GGATCCAGACCTGTTGGAAGA-3' |
| shv0a1-2 | murine *Atp6v0a1* | 5'-GGAAATCACCAGAGCTGATAT-3' |
| shV0A1 | human *ATP6V0A1* | 5'-GGTCCTTCGCCGTCAGTATTT-3' |

**Supplementary Table 3. siRNA sequences and shRNA sequences targeting different genes (continued)**

| **Name** | **Targeted gene** | **Sequence** |
| --- | --- | --- |
| siNr1h3 | murine *Nr1h3* | Sense:5'- GGAGUGUCGACUUCGCAAAUG-3';  Anti-sense:5'- UUUGCGAAGUCGACACUCCUG-3' |
| siNr1h2 | murine *Nr1h2* | Sense:5'- GCUGCUUCGUGACCCACUAUG-3';  Anti-sense:5'- UAGUGGGUCACGAAGCAGCCU-3' |
| SiTGFB1 | human *TGFB1* | Purchased from Guangzhou RiboBio Co., Ltd.  Catalogue number: SIGS0003603-1 |
| siTgfb1-1 | murine *Tgfb1* | Sense:5'- GAAGCGGACUACUAUGCUAAA-3';  Anti-sense:5'- UAGCAUAGUAGUCCGCUUCGG-3' |
| siTgfb1-2 | murine *Tgfb1* | Sense:5'- GCUGCUACUGCAAGUCAGAGA-3';  Anti-sense:5'- UCUGACUUGCAGUAGCAGCGG-3' |

**Note:** shv0a1 in the present manuscript means shv0a1-1 unless indicated otherwise
