# Supplementary material for: ATP6V0A1-dependent cholesterol absorption in colorectal cancer cells triggers immunosuppressive signaling to inactivate memory CD8+ T cells
Source: Nat Commun. 2024 Jul 6;15:5680. doi: 10.1038/s41467-024-50077-7 (PMC11227557; doi:10.1038/s41467-024-50077-7)
Supplement: Supplementary file 3 — Reporting Summary [file 41467_2024_50077_MOESM3_ESM.pdf]

nature portfolio

Corresponding author(s): Li Fu

Last updated by author(s): Jun 12, 2024

Reporting Summary

Nature Portfolio wishes to improve the reproducibility of the work that we publish. This form provides structure for consistency and transparency in reporting. For further information on Nature Portfolio policies, see our Editorial Policies and the Editorial Policy Checklist.

Statistics

For all statistical analyses, confirm that the following items are present in the figure legend, table legend, main text, or Methods section.

n/a	Confirmed	
☐	☒	The exact sample size (*n*) for each experimental group/condition, given as a discrete number and unit of measurement
☐	☒	A statement on whether measurements were taken from distinct samples or whether the same sample was measured repeatedly
☐	☒	The statistical test(s) used AND whether they are one- or two-sided<br>*Only common tests should be described solely by name; describe more complex techniques in the Methods section.*
☒	☐	A description of all covariates tested
☐	☒	A description of any assumptions or corrections, such as tests of normality and adjustment for multiple comparisons
☐	☒	A full description of the statistical parameters including central tendency (e.g. means) or other basic estimates (e.g. regression coefficient) AND variation (e.g. standard deviation) or associated estimates of uncertainty (e.g. confidence intervals)
☐	☒	For null hypothesis testing, the test statistic (e.g. *F*, *t*, *r*) with confidence intervals, effect sizes, degrees of freedom and *P* value noted<br>*Give P values as exact values whenever suitable.*
☒	☐	For Bayesian analysis, information on the choice of priors and Markov chain Monte Carlo settings
☒	☐	For hierarchical and complex designs, identification of the appropriate level for tests and full reporting of outcomes
☐	☒	Estimates of effect sizes (e.g. Cohen's *d*, Pearson's *r*), indicating how they were calculated

Our web collection on statistics for biologists contains articles on many of the points above.

Software and code

Policy information about availability of computer code

Data collection	A description of the software and code has been included in the Methods. In scRNA-seq analysis, raw fastq files were processed with CellRanger V3. In quantitative proteomics analysis, the raw data were searched against Mus_musculus_uniprot_2020_7_2.fasta (86555 sequences) database by Proteome Discoverer 2.4 (Thermo). For flow cytometry assay, the data was collected using the BD FACSAria system (BD Biosciences) or CytoFLEX system (Beckman Coulter).
Data analysis	Data were analyzed using Prism 9.0 (GraphPad Software Inc., La Jolla, CA, USA), R package of KEGGREST (version 1.40.0), or R package of GSVA (Gene Set Variation Analysis). Correlation analysis were performed using Pearson's method. Flow cytometry data was analyzed by FlowJo software (BD Biosciences) and Kaluza software (Beckman Coulter).

For manuscripts utilizing custom algorithms or software that are central to the research but not yet described in published literature, software must be made available to editors and reviewers. We strongly encourage code deposition in a community repository (e.g. GitHub). See the Nature Portfolio guidelines for submitting code & software for further information.

Data

Policy information about availability of data

All manuscripts must include a data availability statement. This statement should provide the following information, where applicable:

- Accession codes, unique identifiers, or web links for publicly available datasets
- A description of any restrictions on data availability
- For clinical datasets or third party data, please ensure that the statement adheres to our policy

Data are available in public open access repositories, directly included in the article, and uploaded as supplementary information by the authors. The scRNA-seq data used in this study are available in the NIH GEO database under accession code GSE238084 [https://www.ncbi.nlm.nih.gov/geo/query/acc.cgi?acc=GSE238084]. The mass spectrometry proteomics data generated in this study have been deposited in the ProteomeXchange database under accession code PXD044010 [https://proteomecentral.proteomexchange.org/cgi/GetDataset?ID=PXD044010].

Research involving human participants, their data, or biological material

Policy information about studies with human participants or human data. See also policy information about sex, gender (identity/presentation), and sexual orientation and race, ethnicity and racism.

Reporting on sex and gender	This study involved blood samples donated by 12 healthy volunteers and 32 paraffin-embedded human CRC tissue samples from Shenzhen People's Hospital. All human subjects provided informed consent, and Institutional Review Board approval was obtained for this study from Shenzhen University and Shenzhen People's Hospital. These participants are half male and half female, and the age range of these participants was 22-68 years.
Reporting on race, ethnicity, or other socially relevant groupings	All the above participants are Asian.
Population characteristics	See above.
Recruitment	Twelve healthy volunteers without cancer diseases were recruited for collecting the blood sample from Shenzhen University in China, according to the protocols approved by the Ethics Committee of the Shenzhen University Medical School. 32 participants with CRC were recruited from Shenzhen People's Hospital in China according to the protocols approved by the Ethics Committee of Shenzhen People's Hospital. The Participants gave informed consent to participate in the study before enrollment.<br>One potential source of bias in the present study is self-selection bias. Participants who chose to enroll in this study may have different characteristics or motivations compared with those who chose not to participate. Another potential source of bias is sampling bias, as our study participants were recruited from Shenzhen People's Hospital in China and may not be representative of other populations or settings. These biases could limit the generalizability of our findings to the broader population of CRC patients. To minimize these biases, we used a standardized protocol for recruitment and data collection, and adjusted the statistical models for potential confounding factors such as age and sex. However, these steps may not completely eliminate the potential for bias; Future research should aim to replicate the present findings in other populations and settings to enhance the generalizability of the results.
Ethics oversight	This study was approved by the Ethics Committee of the Shenzhen University Medical School and that of Shenzhen People's Hospital.

Note that full information on the approval of the study protocol must also be provided in the manuscript.

Field-specific reporting

Please select the one below that is the best fit for your research. If you are not sure, read the appropriate sections before making your selection.

☒ Life sciences ☐ Behavioural & social sciences ☐ Ecological, evolutionary & environmental sciences

For a reference copy of the document with all sections, see nature.com/documents/nr-reporting-summary-flat.pdf

Life sciences study design

All studies must disclose on these points even when the disclosure is negative.

Sample size	Sample size was determined based on statistical power calculations, taking into account the expected effect size, significance level, and statistical power. Relevant guidelines and recommendations from organizations such as EMEA and NCI were consulted in determining sample size.
Data exclusions	No data were excluded from analysis.
Replication	In this study, all experiments were performed independently in triplicate. Each experimental condition was repeated using separate cell cultures or tissue samples three times. By performing experiments in triplicate, we ensured that the results presented in this study were repeatable. Additionally, we performed technical replicates within each experimental condition to ensure that the obtained data were not due to chance variation. At least three technical replicates were performed in this study, and the data for each experimental condition were

	shown as means±s.e.m. The statistics analysis was performed based on these repeated data, and $P < 0.05$ was considered statistically significant.
Randomization	In the animal experiments with gene editing cancer cells, mice are randomly grouped based on their body weight to ensure that the background of mice between different groups is as consistent as possible. In mouse tumor treatment experiments, the criteria for random grouping also need to consider the tumor size of mice to ensure that the initial tumor size in different groups of mice is as consistent as possible.
Blinding	Blinding was not relevant to this research. As a technique usually used in clinical trials to reduce the potential for bias or subjective influence on the outcomes, blinding involves withholding information about the treatment or intervention from the participants or investigators to minimize the potential for placebo effects or experimenter bias. However, in this study, the intervention or exposure was not subjective, and it was impossible to blind the investigators to the exposure. Moreover, the outcomes of interest in our study were objective and measured using standardized methods and instruments, which reduces the potential for subjective interpretation or bias. Therefore, blinding was irrelevant in our research based on the nature of the intervention and outcomes. We used rigorous controls and statistical methods to exclude the potential bias or confounding factors.

Reporting for specific materials, systems and methods

We require information from authors about some types of materials, experimental systems and methods used in many studies. Here, indicate whether each material, system or method listed is relevant to your study. If you are not sure if a list item applies to your research, read the appropriate section before selecting a response.

Materials & experimental systems

n/a	Involved in the study
☐	☒ Antibodies
☐	☒ Eukaryotic cell lines
☒	☐ Palaeontology and archaeology
☐	☒ Animals and other organisms
☒	☐ Clinical data
☒	☐ Dual use research of concern
☒	☐ Plants

Methods

n/a	Involved in the study
☒	☐ ChIP-seq
☐	☒ Flow cytometry
☒	☐ MRI-based neuroimaging

Antibodies

Antibodies used	Rabbit anti-ATP6V0A1: 13828-1-AP, Proteintech Group, Inc;<br>β-actin recombinant antibody: 81115-1-RR, Proteintech Group, Inc;<br>Rabbit anti-RABGEF1: 12735-1-AP, Proteintech Group, Inc;<br>TGF-β1 recombinant antibody (EPR21143): ab215715, Abcam;<br>Ultra-LEAF™ Purified anti-human/mouse TGF-β1 Antibody (19D8): 521707, Biolegend;<br>FITC-conjugated anti-CD8a (53-6.7): eBioscience;<br>PerCP-Cyanine5.5-conjugated anti-CD44 (IM7): eBioscience;<br>PE-conjugated anti-perforin (eBioOMAK-D): eBioscience;<br>APC-conjugated anti-GzmB (QA16A02): BioLegend;<br>PE-conjugated anti- IFN-γ (XMG1.2): eBioscience;<br>BV421-conjugated anti-CD45 (30-F11): BD Horizon™;<br>PerCP-Cyanine5.5-conjugated anti-CD8a (53-6.7): BD Horizon™;<br>APC-conjugated anti-CD44 (IM7): BD Horizon™;<br>PE-conjugated anti-GzmB (QA16A02): BioLegend;<br>BV421-conjugated anti-CD45 (HI30): BD Horizon™;<br>FITC-conjugated anti-CD8a (RPA-T8): eBioscience;<br>APC-conjugated anti-CD45RO (UCHL1): BioLegend;<br>PerCP-Cyanine5.5-conjugated anti-IFN-γ (4S.B3): BioLegend);<br>PE-conjugated anti-GzmB (QA16A02): BioLegend;<br>Rab7 (D95F2) XP® Rabbit mAb: 9367, CST;<br>VPS41 mouse mAb (D-12): sc-377118, Santa Cruz Biotechnology;<br>LAMP1 mouse mAb (H4A3): sc-20011, Santa Cruz Biotechnology.
Validation	According to the corresponding manufacturer's websites, rabbit anti-ATP6V0A1 (13828-1-AP, Proteintech Group), β-actin recombinant antibody (81115-1-RR, Proteintech Group), rabbit anti-RABGEF1 (12735-1-AP, Proteintech Group),and TGF-β1 recombinant antibody (EPR21143, Abcam) have been validated for WB and IHC assay in human and mouse samples. Ultra-LEAF™ Purified anti-human/mouse TGF-β1 Antibody (19D8, Biolegend) was validated for its function of blocking human/mouse TGF-β1 by the manufacturer. Rab7 (D95F2) XP® Rabbit mAb, VPS41 mouse mAb (D-12), and LAMP1 mouse mAb (H4A3) have been validated for the IF application against human/mouse samples as described in the manufacturer's websites. FITC-conjugated anti-CD8a (53-6.7), PerCP-Cyanine5.5-conjugated anti-CD44 (IM7), PE-conjugated anti-perforin (eBioOMAK-D), APC-conjugated anti-GzmB (QA16A02), and PE-conjugated anti- IFN-γ (XMG1.2) were validated by the manufactures for the flow cytometry detection in mouse samples. As described in the manufacturer's websites, FITC-conjugated anti-CD8a (RPA-T8), APC-conjugated anti-CD45RO (UCHL1), PerCP-Cyanine5.5-conjugated anti-IFN-γ (4S.B3), and PE-conjugated anti-GzmB (QA16A02), have been validated for their application of

detecting human samples with flow cytometry. We also confirmed the specificity and sensitivity of each antibody using positive and negative control samples and verified their optimal dilution through titration experiments

Eukaryotic cell lines

Policy information about cell lines and Sex and Gender in Research

Cell line source(s)	MC38, CT26, HCT-8 cells; The cell lines were obtained from ATCC.
Authentication	Short tandem repeat (STR) profiling analysis was used for cell line authentication.
Mycoplasma contamination	All the cell lines used in this study were tested negative for mycoplasma.
Commonly misidentified lines (See ICLAC register)	No commonly misidentified lines were used in this study.

Animals and other research organisms

Policy information about studies involving animals; ARRIVE guidelines recommended for reporting animal research, and Sex and Gender in Research

Laboratory animals	Four- to six-week-old female C57BL/6J mice, BALB/c mice, C57BL/6 Rag2-/-Il2rg-/- mice, BRG mice, and NOD.CB17-Prkdcscid/NcrCrl (NOD/SCID) mice were purchased from Charles River Laboratories (Beijing, China) or Shanghai Model Organisms Center, Inc. (Shanghai, China), and four- to six-week-old female NOD/ShiLtJGpt-Prkdcem26Cd52Il2rgem26Cd22/Gpt (NCG) mice were purchased from Gempharmatech Co., Ltd (Nanjing, China). The mice were maintained under specific pathogen-free conditions and cohoused with five mice per incubator with a 12-hour light/dark cycle.
Wild animals	No wild animals were used in this study.
Reporting on sex	The findings do not only apply to one sex or gender. Sex and gender-based information was not collected in the animal experiments here.
Field-collected samples	No field-collected samples were used.
Ethics oversight	The animal study protocol was approved by Institutional Animal Care and Use Committee at Shenzhen University Medical School.

Note that full information on the approval of the study protocol must also be provided in the manuscript.

Plants

Seed stocks	No plants were used in this study.
Novel plant genotypes	No plants were used in this study.
Authentication	No plants were used in this study.

Flow Cytometry

Plots

Confirm that:

- [x] The axis labels state the marker and fluorochrome used (e.g. CD4-FITC).
- [x] The axis scales are clearly visible. Include numbers along axes only for bottom left plot of group (a 'group' is an analysis of identical markers).
- [x] All plots are contour plots with outliers or pseudocolor plots.
- [x] A numerical value for number of cells or percentage (with statistics) is provided.

Methodology

Sample preparation	For the in vitro T-cell stimulation analysis, CD8+ T cells were isolated from C57BL/6J mice-bearing MC38 wild-type tumors using a negative mouse CD8+ T-cell isolation kit (STEMCELL). These isolated CD8+ T cells were cultured in RPMI 1640 medium containing 10% FBS with IL-2 (10 ng/mL) and treated with the following: SMAD3 inhibitor (E)-SIS3, MC38 cell culture medium plus control IgG or anti-TGF-β1, conditioned medium (CM) from MC38-shNTC cells or MC38-shv0a1 cells with/without

	incubation with recombinant mouse TGF-β1 protein. Brefeldin A (eBioscience) was added to the culture in the last 4 hours, and the CD8+T cells were analyzed after the staining with antibodies. In another experiment to directly detect memory T-cell activities, TILs were isolated from C57BL/6J mice-bearing MC38 shNTC or shv0a1 tumors and directed to the antigen staining and flow cytometry analysis.
Instrument	The stained cells were analyzed using the BD FACSAria system (BD Biosciences) or the CytoFLEX system (Beckman Coulter).
Software	Kaluza (Beckman) and Treestar FlowJo (v10.8.1) software were used to analyze flow cytometry data.
Cell population abundance	To analyze the level of different stained antigens, the intact cells was firstly required to be gated, and the dots representing intact cells usually account for over 95% of all dots in the FSC/SSC dot plots. Usually, the single cells account for over 95% of all intact cells as the FSC-H/FSC-A dot plots. Live/dead dyes and anti-CD45 staining were then used to gate the viable immune cells which may account for ~40% of the single cells.
Gating strategy	As described in the manuscript (Figure 3D), all the intact cells were gated based on the FSC/SSC dot plots to exclude the cell debris, and the single cells without condensation were gated from intact cells based on the FSC-H/FSC-A dot plots. Live/dead dyes and anti-CD45 staining were then used to gate the viable immune cells. The indicated staining-positive cells were then gated from the viable immune cells and were further analyzed for cytokine expression. The boundary between staining negative cells and positive cells was defined according to the corresponding negative and positive controls.

☒ Tick this box to confirm that a figure exemplifying the gating strategy is provided in the Supplementary Information.
