# Supplementary material for: ATP6V0A1-dependent cholesterol absorption in colorectal cancer cells triggers immunosuppressive signaling to inactivate memory CD8+ T cells
Source: Nat Commun. 2024 Jul 6;15:5680. doi: 10.1038/s41467-024-50077-7 (PMC11227557; doi:10.1038/s41467-024-50077-7)
Supplement: Supplementary file 4 — Inventory of Supporting Information [file 41467_2024_50077_MOESM4_ESM.docx]

**Inventory of Supporting Information**

Manuscript #: **NCOMMS-23-46893B**

Corresponding author name(s): Li Fu

The Supporting Information items include the following sections:

● Supplementary Information file

-Supplementary Figures 1-26 and their figure legends;

-Supplementary Tables 1-3.

● Source Data file: An excel document; The relevant raw data from each figure is represented by a single sheet in this document.
